# Model Lipid Membranes Assembled from Natural Plant Thylakoids into 2D Microarray Patterns as a Platform to Assess the Organization and Photophysics of Light‐Harvesting Proteins

**DOI:** 10.1002/smll.202006608

**Published:** 2021-03-09

**Authors:** Sophie A. Meredith, Takuro Yoneda, Ashley M. Hancock, Simon D. Connell, Stephen D. Evans, Kenichi Morigaki, Peter G. Adams

**Affiliations:** ^1^ School of Physics and Astronomy and The Astbury Centre for Structural Molecular Biology University of Leeds Leeds LS2 9JT UK; ^2^ Graduate School of Agricultural Science and Biosignal Research Center Kobe University Rokkodaicho 1‐1, Nada Kobe 657‐8501 Japan

**Keywords:** artificial photosynthesis, atomic force microscopy (AFM), biohybrids, chlorophyll fluorescence, fluorescence lifetime imaging microscopy (FLIM), light‐harvesting, supported lipid bilayers

## Abstract

Natural photosynthetic “thylakoid” membranes found in green plants contain a large network of light‐harvesting (LH) protein complexes. Rearrangement of this photosynthetic machinery, laterally within stacked membranes called “grana”, alters protein–protein interactions leading to changes in the energy balance within the system. Preparation of an experimentally accessible model system that allows the detailed investigation of these complex interactions can be achieved by interfacing thylakoid membranes and synthetic lipids into a template comprised of polymerized lipids in a 2D microarray pattern on glass surfaces. This paper uses this system to interrogate the behavior of LH proteins at the micro‐ and nanoscale and assesses the efficacy of this model. A combination of fluorescence lifetime imaging and atomic force microscopy reveals the differences in photophysical state and lateral organization between native thylakoid and hybrid membranes, the mechanism of LH protein incorporation into the developing hybrid membranes, and the nanoscale structure of the system. The resulting model system within each corral is a high‐quality supported lipid bilayer that incorporates laterally mobile LH proteins. Photosynthetic activity is assessed in the hybrid membranes versus proteoliposomes, revealing that commonly used photochemical assays to test the electron transfer activity of photosystem II may actually produce false‐positive results.

## Graphical abstract

A polymerized lipid bilayer (purple) templates the self‐assembly of “hybrid membranes” from thylakoid membranes (green) and synthetic lipids (colorless). During membrane formation, transmembrane proteins migrate from thylakoids into underlying lipid bilayers, decreasing the protein concentration and uncoupling protein‐protein interactions (manifested as increased fluorescent lifetimes). These hybrid membranes are used to delineate the relationship between photophysics and structure of light‐harvesting complexes.

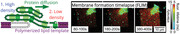

## 1 Introduction

**Figure 1:**
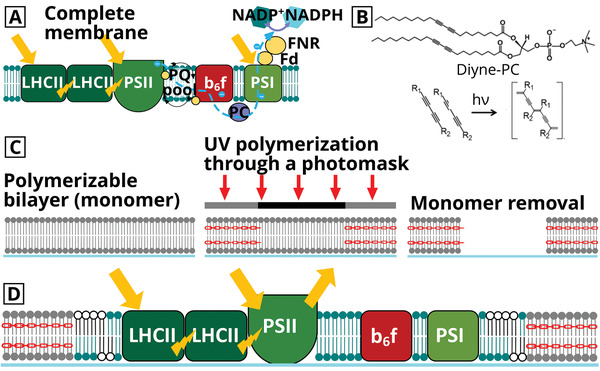
Concepts for designing model LH membranes, as reported recently.^[^ ^17^ ^]^ a) Schematic of the natural thylakoid membranes and the energy transfer processes occurring. Yellow arrows represent absorption of light, yellow bolts represent interprotein excitation energy transfer, and blue dashed lines represent the electron transfer chain (simplified). b) Chemical structure of the Diyne‐PC and the photopolymerization reaction. c) Schematic of the how photopolymerization is carried out through a photomask to generate array patterns, where only the regions of Diyne‐PC exposed to UV become crosslinked (red linkers indicate polymerized lipids). d) Schematic of the “hybrid membranes” within the polymer‐lipid template.

The absorption of solar energy and subsequent transduction to chemical energy in the early stages of photosynthesis has a quantum efficency approaching unity.^[^ ^1^ ^]^ This efficiency and the ability of photosynthetic biomolecules to participate in electronic circuits^[^ ^2^ ^]^ makes them possible candidates for the development of photonic biohybrid nanotechnologies, e.g., photo‐biosensors^[^ ^3^ ^]^ and optoelectronics.^[^ ^4^ ^]^ The natural photosynthetic membranes of green plants are found within chloroplasts and are termed “thylakoid membranes.” These membranes contain a large network of light‐absorbing proteins, each protein containing a high density of pigments. Photon absorption by a pigment molecule (e.g., chlorophyll, carotenoid) produces an excited electronic state which can be transferred with high efficiency between the pigments within one protein and between nearby proteins. In plants, many copies of the protein light‐harvesting complex II (LHCII) form a large “antenna” system that performs rapid and highly efficient transfer of excitation energy to photosystem (PS) proteins, which then perform photochemistry.^[^ ^5^ ^]^ Photochemistry in the PSII leads to a cycle of electron transport through the membrane, coupled to the unidirectional pumping of protons across the membrane, using several intermediate proton and electron carriers (**Figure** **1**a). This proton gradient is a temporary store of chemical and electrical energy, which can be harnessed to generate high‐energy biomolecules (e.g., adenosine triphosphate and NADPH).^[^ ^6^ ^]^

The interaction between the photosynthetic machinery relies heavily on protein arrangement and the surrounding superstructure of the thylakoid lipid membrane.^[^ ^7^ ^]^ In the native system, LHCII and PSII are organized into “supercomplexes” which are located within stacked membranes, called “grana.”^[^ ^8^ ^]^ The overall stacked membrane arrangement provides a large surface area for incorporating many hundreds of pigments across >100 nm, creating a wide spatial and optical cross section for the absorption of sunlight. The main antenna protein, LHCII, undergoes concentration‐dependant quenching apparently based on the extent of LHCII–LHCII associations. Large‐scale rearrangements of LHCII and PSII within grana can change the extent of protein–protein interactions, modulating the energy balance within the system and opening/closing additional energy dissipation pathways.^[^ ^9^ ^]^ This effect is observed in spectroscopy of model systems (e.g., LHCII aggregates and proteoliposomes) and natural membranes (e.g., chloroplasts and plant leaves) as a descreased fluorescence intensity and shorter fluorescence lifetime, often termed “fluorescence quenching.”

Determining the concentration and relative positions of LH and PS proteins is therefore crucial for understanding both excitation energy migration and electron transport across membranes.^[^ ^10^ ^]^ Electron microscopy has been important for revealing the superstructure of photosynthetic membranes and the positions of proteins within membranes,^[^ ^11^ ^]^ however, it is time‐consuming, expensive, and cannot usually be performed on hydrated samples at room temperature. In contrast, atomic force microscopy (AFM) allows the visualization of LH and PS protein complexes at relatively high resolution (≈1 nm laterally and ≈0.1 nm vertically) and can measure membrane samples under close‐to‐native conditions (ambient temperature, aqueous environment, etc.).^[^ ^12^ ^]^ Depositing thylakoid membranes which have been extracted from chloroplasts onto a flat, solid surface such as mica or glass can allow high‐resolution AFM and spectroscopy to be performed and increase our understanding of the interactions within the native system. However, isolated and fragmented natural membranes are not necessarily an ideal platform to assess system functionality because of their heterogeneous composition and unstable nature.

The photophysical properties of LH proteins have also been studied using nanoscale array patterns of proteins on solid surfaces^[^ ^13^ ^]^ and LH proteins incorporated into model membranes (proteoliposomes).^[^ ^9^, ^14^ ^]^ These model systems offer several advantages as platforms to study the inherent physicochemical properties of the proteins, such as providing precise control over protein arrangement, known membrane composition, and the incorporation of specific lipids to help maintain protein stability.^[^ ^14^, ^15^ ^]^ However, many of these models require extensive biochemical purification, chemical alteration of the protein or the support surface, and/or removal of the native lipids, any of which may affect the stability and photophysical state of LH and PS proteins.^[^ ^14^, ^16^ ^]^ Furthermore, these models are often limited to one or two types of protein which simplifies the complex interactions that are present in the native system, because of the procedural challenge of reconsituting multiple types of purified protein into a single artificial lipid membrane.

Therefore, there is a compelling need for a thylakoid membrane model that has an intermediate level of complexity: consisting of the full range of proteins found in the native thylakoid membrane but with a greater control over membrane composition and amenability to high‐resolution microscopy (i.e., surface based). An ideal model system for the study of photosynthetic membranes would consist of a stable membrane on a solid support which contains the complete network of photosynthetic proteins embedded within a bilayer comprised of a native‐like mixture of lipids. Very recently, Morigaki and co‐workers presented a solution through a new type of “hybrid membranes” by incorporating thylakoid components into supported lipid bilayers (SLBs) within an array‐patterned template.^[^ ^17^ ^]^ The empty templates were formed from photopolymerized diacetylene‐phosphocholine (Diyne‐PC) lipids and have exposed lipid bilayer edges (Figure 1b,c), which promote the formation of hybrid membranes from the combination of synthetic lipid vesicles and natural thylakoid membrane (Figure 1d).^[^ ^18^ ^]^ The result is an array of discrete, high‐quality SLBs that are patterned into easily recognizable microarrays to allow for more accurate analysis.^[^ ^19^ ^]^

These hybrid membranes could provide a model system to understand the photophysical and biochemical processes of photosynthesis and to inspire the design of new nanotechnologies.^[^ ^20^ ^]^ A crucial next step is to understand the structural arrangement and diffusivity of the thylakoid proteins (and lipids) and to apply this model system as a platform to assess their photophysical state. Specific to the photophysics, it would be useful to determine the fluorescence lifetime within the system because this represents the stability of the chlorophyll excited states, a crucial parameter related to the propensity for energy transfer or dissipation. However, the initial characterization performed on this model^[^ ^17^ ^]^ used simple epifluorescence microscopy to visualize the membranes at microscale resolution and did not resolve information about the nanoscale membrane structure or photophysical state of the system.

Here, we present a quantitative characterization of the nanoscale structure and photophysical properties of photosynthetic hybrid membrane using fluorescence lifetime imaging microscopy and atomic force microscopy. To test the efficacy of this platform, we address the following questions: i) What is the nanoscale structure of the hybrid membranes compared to natural thylakoid membranes? ii) How does the fluorescence lifetime of the hybrid membranes compare to the natural thylakoid membranes? iii) What is the protein concentration of the hybrid membranes relative to the native system? iv) How do thylakoid proteins diffuse and reorganize within the membrane? v) Can the platform be used for more effective functionality assays of the photosynthetic activity (electron transport)? In addition to characterizing the final form of the hybrid membranes, we visualize the membrane formation to measure the migration of lipids and proteins in real time.

## 2 Results and Discussion

### 2.1 Comparing the Structure and Photophysical State of Natural Thylakoid Membranes versus Hybrid Membranes Using Fluorescence Lifetime Imaging Microscopy and Atomic Force Microscopy

Tightly‐packed LH proteins are known to have relatively short fluorescence lifetimes compared to isolated LH proteins owing to these protein–protein interactions; therefore, changes to the protein arrangement will alter the degree of fluorescence quenching^[^ ^9d,g^ ^]^ To assess interactions between LH proteins, we used fluorescence lifetime measurements which quantify the degree of quenching (from the excited state decay rate).^[^ ^21^ ^]^ Specifically, a laser‐scanning fluorescence microscope was used to acquire images (at ≈300 nm resolution) where each pixel has both fluorescence intensity and a time‐resolved fluorescence spectrum, termed “fluorescence lifetime imaging microscopy” (FLIM).^[^ ^9^, ^13^ ^]^ The fluorescence lifetime, as determined via single‐photon measurements of the pico/nanosecond timescale of excited state decay, is consistent for isolated proteins and only changes if proteins undergo molecular interactions or energy transfer. Therefore, we can use this parameter to represent the photophysical state of LH and PS proteins and to ascertain dynamic behaviors and protein interactions that occur at length‐scales below the optical resolution of the FLIM. This strategy was used throughout the study to observe the structural arrangement of our membrane samples correlated to their photophysical properties.

**Figure 2:**
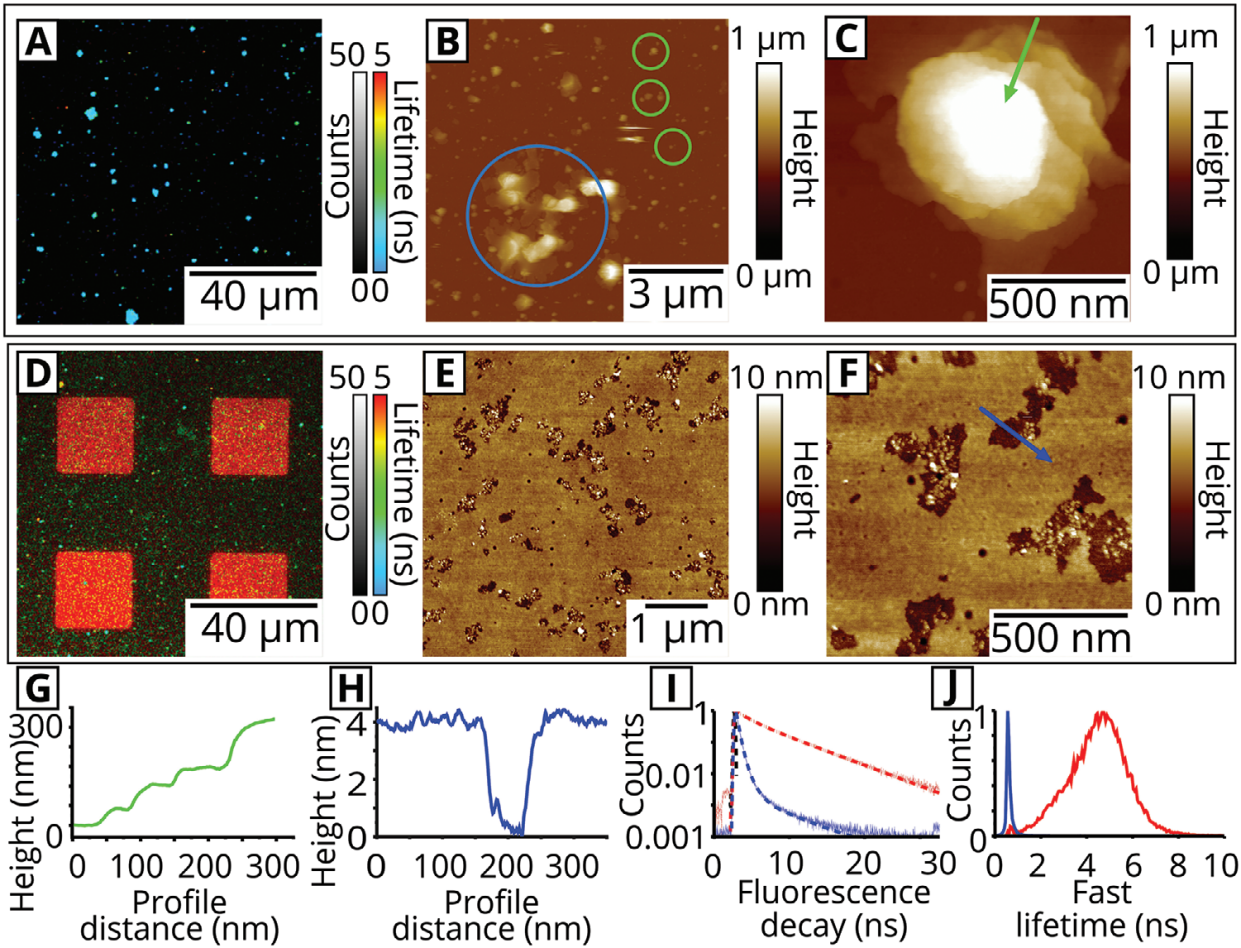
Analysis of extracted thylakoids versus hybrid membranes via FLIM. All were incubated for 30 min and then washed with aqueous buffer. All images show selective excitation of chlorophyll at 485 nm and collection of fluorescence emission between 655 and 725 nm. a) FLIM image of extracted thylakoid membranes adhered onto a hydrophilic glass surface. b) AFM image of a similar sample as in (a). The topograph shows small, adhered membrane patches (ringed green) and larger multilayered structures (ringed blue). c) A zoomed‐in topograph of a multilayered thylakoid extract. d) FLIM image of the “hybrid membranes” showing Chl fluorescence with a long (red) lifetime localized in the square regions of a Diyne‐PC template. Smaller short (blue) lifetime particles are present across the field of view, suspected to be loosely adsorbed thylakoids. e) AFM topographic image of a similar sample as in (d). f) A zoomed‐in topograph of the hybrid membrane. g) A height profile drawn along the green line in panel (c), showing the multilayer steps in a thylakoid extract. h) A height profile drawn along the blue line shown in panel (f), showing the step height across a pore in the hybrid membrane. i) Normalized fluorescence decay curves: showing raw data (pale lines) and fits (dashed lines) on log/linear axes (*y*/*x*). The fluorescence decay curve for intact thylakoids (blue) in image (a) decays more rapidly than the decay curve for hybrid membranes (red) in (d) (*N* = 16 corrals). j) Frequency distribution of the “FastFLIM” fluorescence lifetime (binned over 25 ps), samples colored as in (i), normalized to a peak of 1. Extracted thylakoids (blue) have a significantly narrower distribution than hybrid membranes (red).

First, a type of natural photosynthetic membranes was analyzed as a baseline to compare to our model system. Thylakoid membranes were extracted from plant chloroplasts following standard protocols, and a basic spectroscopy characterization confirmed that these samples contained the expected proteins (LHCII, PSII, PSI, see Figure S1, Supporting Information). To assess the initial photophysical state and structure of the natural biomembranes, these “extracted thylakoids” were adhered to a hydrophilic glass coverslip to make them accessible to imaging via FLIM and AFM. **Figure** **2**a shows a representative FLIM image obtained from the chlorophyll (Chl) fluorescence of extracted thylakoids, revealing many distinct objects which all appear to have similar, quite short fluorescence lifetimes of ≈0.5 ns. Note, all FLIM images have a color scale with fluorescence lifetime represented from blue (short lifetime) to red (long lifetime) and an intensity scale representing the total counts in each pixel. Control measurements show that there is minimal nonspecific fluorescence from any other sources or impurities (Figure S2, Supporting Information), so we can be confident in the detection of chlorophyll fluorescence even where the signal is low.

Topographical maps of the thylakoids adhered to glass, provided by AFM, reveal that these objects have a heterogeneous size distribution. In a large field of view (Figure 2b), a variety of structures are observed, from relatively compact assemblies (100–200 nm laterally and 10–100 nm in height, ringed green) to large microscale structures (3–4 µm laterally and up to 750 nm high, ringed blue) which contain distinct multilayers. Figure 2c,g shows a multilayered structure, where a height profile (green) has been drawn to show the increase in height over consecutive multilayers, up to ≈300 nm above the underlying substrate. PSII has a crystallographic height of ≈10 nm,^[^ ^12b^ ^]^ so it is likely that even the smallest objects observed via AFM must consist of a few stacked protein‐rich membranes, increasing up to tens of stacked membranes for the largest objects. The structures observed by AFM are consistent with the tightly stacked thylakoid grana membranes observed in vivo, and the short fluorescence lifetime is also in agreement with that typically measured for native thylakoid membranes. The fact that such a heterogeneous and disordered distribution of randomly adhered membranes is observed, highlights the need for a method to promote the formation of large, homogeneous membrane structures that are suitable for quantitative studies and light‐harvesting nanotechnologies.

To analyze the Chl signal of thylakoid extracts quantitatively, a fluorescence decay curve was generated by accumulating the photons collected from the whole image (Figure 2i, blue curve): this reveals a mean fluorescence lifetime 〈τ〉 = 0.40 ± 0.01 ns (*N* > 500 particles ± standard deviation). This lifetime is in agreement with our ensemble spectroscopy data of the extracted thylakoids in solution (〈τ〉 ≈ 0.5 ns) and is in good agreement with previous reports of LHCII and PSII within intact chloroplasts and leaves.^[^ ^22^ ^]^ This 〈τ〉 is much shorter than the lifetime known for isolated LH and PS proteins in detergent (≈4 ns), as expected, owing to the quenching effect of protein–protein interactions present in thylakoids. Overall, this shows that our FLIM data on LH membrane samples agree nicely with standard spectroscopy.

Hybrid membranes were prepared in a two‐stage process. In stage 1, templates of polymerized Diyne‐PC on glass coverslips were generated by photolithography, as previously published^[^ ^17^ ^]^ and shown schematically in Figure 1c. The photolithogaphy generates a pattern based on the design of the photomask used; here, we chose a square‐array grid pattern expected to produce an array of lipid bilayers with exposed edges providing large 20 × 20 µm corral regions of empty glass (Figure S3, Supporting Information). In stage 2, natural and synthetic membranes were combined to fill the empty regions of the template and fuse with the exposed edges to form a corraled SLB. Specifically, Diyne‐PC templates were incubated with an aqueous suspension of extracted thylakoids and synthetic lipid vesicles (1,2‐dioleoyl‐*sn*‐glycero‐3‐phosphocholine, DOPC) in a 1:3 weight/weight ratio, and the sample was washed with clean buffer solution. FLIM performed on these samples of “hybrid membranes” is shown in Figure 2d, revealing clear array patterns where the vast majority of Chl fluorescence is localized within the square corral regions defined by the template. These patterned hybrid membranes were highly reproducible, with similar dimensions and fluorescence intensity across multiple preparations (Figure S4, Supporting Information). High‐resolution AFM topographs (Figure 2e,f) taken at the center of the hybrid membrane reveal the structure to be mostly flat and homogeneous across widespread areas. Multiple small pores were present in the membrane and occupy ≈10% of measured area. These pores typically have a lateral scale of ≈100 nm and an average depth of 4.45 ± 0.62 nm (*n* = 10 profiles, Figure 2h). The depth of these pores is in agreement with the published values for a DOPC bilayer (≈4.5 nm) and show that the hybrid membranes have a similar thickness.^[^ ^23^ ^]^ To improve our hybrid membranes in future work, it may be possible to “heal” such defects in lipid bilayers^[^ ^24^ ^]^ to provide increased membrane continuity, by a secondary addition of lipid vesicles which may spread into the pores and merge with existing lipid bilayers.^[^ ^19c^ ^]^ In later sections, we propose mechanisms for the formation of the hybrid membranes and the resulting nanoscale structures that are observed.

The Chl fluorescence lifetime, as determined from analysis of the time‐resolved aspect of the FLIM data, was much longer for these hybrid membranes compared to extracted thylakoids. The fluorescence decay curve generated from analyzing the photons accumulated from the box regions confirmed a slow decay process for hybrid membranes with 〈τ〉 = 4.06 ± 0.11 ns (red curve in Figure 2i). This value represents entirely “non‐quenched” proteins (isolated LHCII in detergent has 〈τ〉 ≈ 4 ns),^[^ ^25^ ^]^ in stark contrast to the short lifetime of extracted thylakoids. The long average lifetime suggests that the protein density in hybrid membranes must be sufficicently low that the protein–protein interactions found in the natural thylakoids (which reduce the fluorescence lifetime as discussed above) are relatively rare. Note, this long fluorescence lifetime was mainly observed inside of the corral region, with a minor subpopulation of small particles with shorter lifetimes (occasional blue/green specks) observed on the surrounding framework. These short‐lifetime particles are likely to be extracted thylakoids that have adhered to the top of the template and not merged with the synthetic lipid bilayers.

It is informative to assess the distribution of lifetimes within each sample, because this allows us to comment on the range of photophysical states within each membrane, rather than merely the average. To do this, a frequency distribution plot of fluorescence lifetime was generated for both samples by binning photons into appropriate time ranges, shown in Figure 2j. These distributions can be fit to Gaussian functions centered around 0.57 and 4.58 ns for the extracted thylakoid sample and the hybrid membrane sample, respectively. The width of the distribution was significantly narrower for extracted thylakoids than hybrid membranes (FWHM_thylakoid_ = 0.15 ns vs FWHM_hybrid_ = 2.31 ns). The broad distribution of lifetimes in the hybrid membranes suggests a variety of photophysical states of the LH and PS proteins, which could be caused by heterogeneity in the protein density or local density fluctuations. The dramatic increase in Chl fluorescence lifetime observed in both the fitted lifetime 〈τ〉 and frequency distributions leads us to conclude that large‐scale protein and lipid reorganizations occur during the hybrid membrane assembly. We hypothesize that the photosynthetic proteins become diluted significantly when thylakoid membranes merge with DOPC lipid bilayers, resulting in a significantly slower rate of energy dissipation and potentially a higher proportion of absorbed energy being re‐emitted as fluorescence, leading to longer fluorescence lifetimes.

To test this hypothesis, we attempted to quantify the change in protein density by careful analysis of the absolute magnitude of fluorescence emission between samples. The fluorescence intensity of hybrid membranes was compared to the fluorescence intensity of control samples containing a known amount of photosynthetic proteins, while taking into account changes in the level of quenching and keeping consistent acquisition parameters. A control sample of “LHCII proteoliposomes” was prepared from a defined quantity of purified LHCII and natural thylakoid lipids, as previously described.^[^ ^14f^ ^]^ The density of proteins in hybrid membranes was then calculated in terms of “LHCII equivalents” in a three‐stage calculation process. First, the approximate number of counts per LHCII proteoliposome was calculated based upon FLIM measurements (Figure S5 and Table S3, Supporting Information). Second, the lipid/protein ratio was calculated from bulk spectroscopy and the estimated molecular packing (Table S4, Supporting Information). Third, the number of LHCII equivalents per corral of hybrid membranes was calculated, based on FLIM data and the values from stages 1 to 2 (Table S5, Supporting Information). Our best estimate for the protein content is 57 100 ± 5500 LHCII equivalents per corral (3 080 000 chlorophylls). Taking into account uncertainities, we estimate a possible range for the protein density of 84–451 LHCII µm^−2^ corresponding to 0.32–3.54% of the total membrane area being occupied by photosynthetic proteins (best estimates of 143 LHCII µm^−2^ and 0.72%). Given that natural photosynthetic membranes are comprised of 60–70% protein by weight,^[^ ^8^ ^]^ these estimates are in agreement with our hypothesis that the hybrid membranes contain a relatively low concentration of proteins. In later sections, we assess if the apparent change in the photophysical state and reduced density affects the functionality and energy transfer within the system.

**Figure 3:**
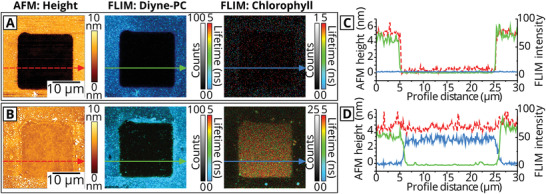
Analysis of hybrid membranes by correlated FLIM and AFM measurements. a,b) The left panel is an AFM topograph, the center panel is the “Diyne‐PC FLIM channel” (i.e., optimized to detect the polymerized lipid by using excitation at 485 nm and collection of emission between 505 and 535 nm), and the right panel is the “Chl FLIM channel” (i.e., optimized to detect the chlorophyll fluorescence from LH and PS proteins by using excitation at 640 nm and collection of emission between 672 and 696 nm). a) Correlated FLIM+AFM data showing a single square of the polymerized lipid “empty” template. The minimal signal in the Chl FLIM channel is statistically indistinguishable from detector noise. b) Correlated FLIM+AFM data showing a similar region as in (a), but after the corrals were “backfilled” with the extracted thylakoids and DOPC liposomes to form the hybrid membrane. c,d) Profiles drawn across the region in (a) or (b), respectively, showing the AFM height (red, dashed), FLIM intensity from Diyne‐PC (green), and FLIM intensity from Chl (blue). The Chl intensity is displayed after multiplication by a factor of 3, for comparison purposes. Higher magnification FLIM+AFM data are shown in Figure S6 (Supporting Information).

To confirm that the hybrid membrane structure observed via AFM in Figure 2e,f was indeed correlated to the areas of Chl fluorescence, an instrument combining AFM with FLIM was used to record nanoscale topography maps spatially correlated to multichannel fluorescence data. The spectral and temporal selectivity of the FLIM allowed us to define two separate FLIM channels: i) the “Chl channel” defined as the combination of selective Chl excitation and a detector optimized for Chl detection, ii) the “Diyne‐PC channel” optimized for the excitation and emission of the intrinsic fluorescence of the polymerized lipid template (note, the spectral overlap between the two fluorescence channels was negligible, see Figure S2 and Table S1, Supporting Information). These two fluorescence channels were probed simultaneously using a pulse‐interleaved excitation mode, with AFM topographs acquired on the same region immediately after, images shown in **Figure** **3**a,b. The AFM height profile in Figure 3c (red line) revealed a 4.81 ± 0.07 nm height from the polymerized lipids to the base of the empty corral, in excellent alignment with the fluorescence intensity profile which drops from ≈75 to ≈0 counts over the same region (Figure 3c, green line). For the “empty” lipid template, the background signal in the Chl FLIM channel was ≈0 counts across the entire image, as expected (Figure 3c, blue line). After the formation of the hybrid membranes, there was largely homogeneous Chl fluorescence within the square corral regions with no resolvable defects at this scale. The increase in the Chl fluorescence intensity (Figure 3d, blue line) corresponded with a step change in the AFM height to a mere 0.19 ± 0.08 nm (Figure 3d, red line). Thus, the average measured thickness of the hybrid membrane was inferred to be 4.62 ± 0.15 nm, in agreement with measurements of the hybrid membrane thickness in Figure 2h. The precise spatial correlation between Chl fluorescence and the topography of the deposited membrane demonstrates that the photosynthetic proteins present specifically within the corral regions and are excluded from the template grid.

### 2.2 Observing the Dynamics of Hybrid Membrane Formation Using Time‐Lapse FLIM

**Figure 4:**
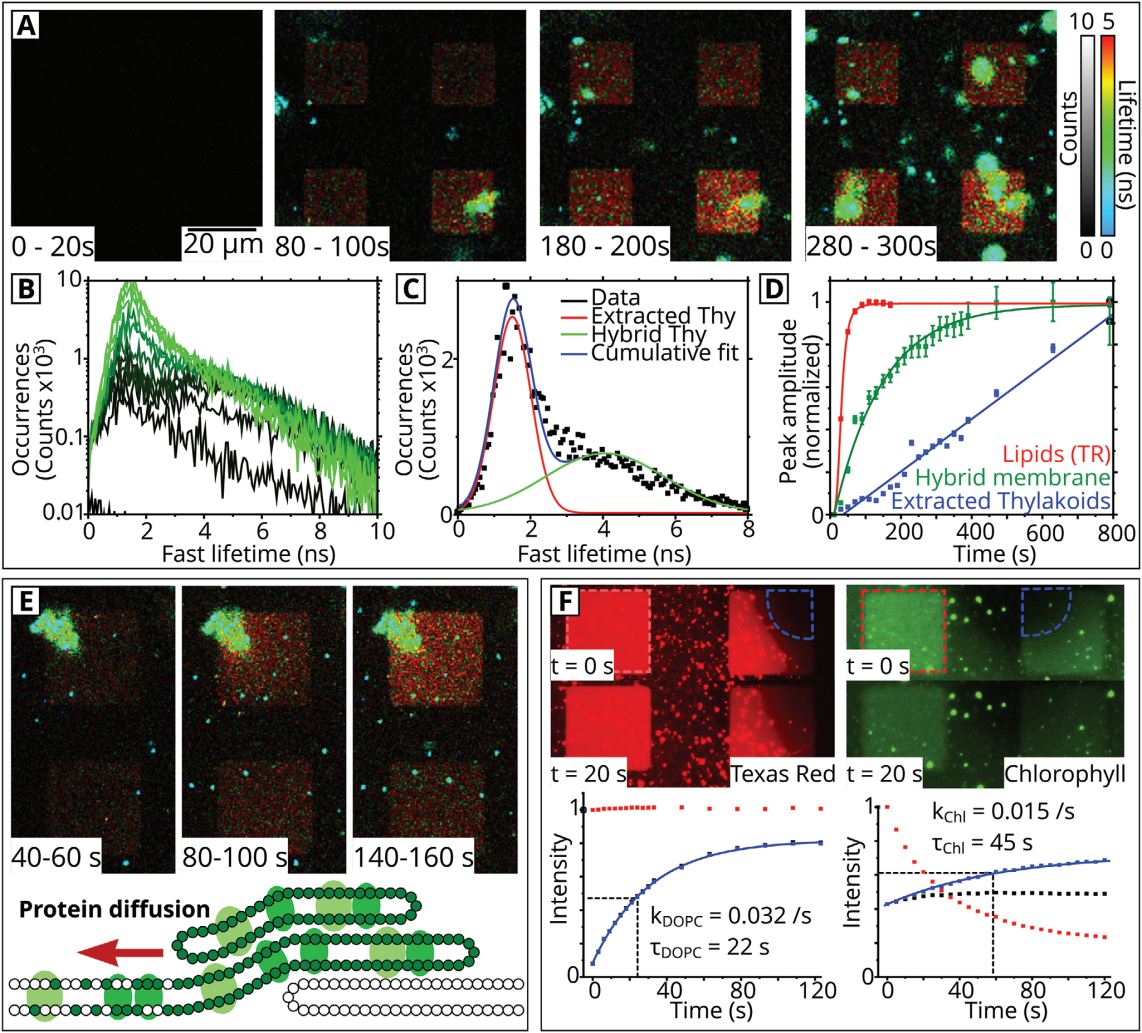
Analysis of the dynamic assembly processes occurring during hybrid membrane formation. a) Time‐lapse series of FLIM images during the formation of hybrid membranes. Each panel shows a 20 s acquisition during the real‐time membrane assembly (using the same excitation and emission optics as in Figure 2). See Videos S1 and S2 (Supporting Information). b) Evolution of the frequency distribution of fluorescence lifetimes during the time series shown in (a). Dark green to light green colored curves represent the lifetime frequency distribution at increasing time‐points. c) An example frequency distribution from a 20 s snapshot (*t* = 220–240 s), deconvoluted into two peaks (long lifetime and short lifetime). d) Analysis of the growth of components of the hybrid membrane over time (normalized to its maximum value for display purposes). Green: photosynthetic proteins in the hybrid membrane, tracked through the long‐lifetime peak amplitude from (c). Blue: extracted thylakoids signal, tracked through the short‐lifetime peak amplitude from (c). Red: lipid accumulation, tracked through fluorescent lipids in a control sample (Figure S8, Supporting Information). e) FLIM timelapse of a thylakoid membrane extract adhered to the top of a developing SLB during membrane formation, proteins diffuse outwardly from the extract over time. Cartoon: Proposed schematic for the self‐assembly of hybrid membranes. f) FRAP experiments to calculate the mobility of proteins (Chl fluorescence) compared to lipids (Texas Red fluorescence). The rate of fluorescence recovery (blue datapoints) was calculated by measuring the intensity of the bleached area (blue dashed region) and correcting the raw recovery data (black datapoint) by the extent of photobleaching in a reference corral (red dashed region, red datapoints).

For our photosynthetic model, understanding the membrane formation process would be useful for explaining the changes in photophysics of the incorporated proteins and the occurrence of micro/nanoscale topographical features. In a biophysical context, one may also wish to understand more about the inherent diffusivity of these proteins. So, the kinetics of protein insertion into the hybrid membranes were studied in real time using time‐lapse FLIM. The intensity and lifetime of Chl fluorescence during membrane assembly over sequential FLIM images are shown in **Figure** **4**a (brightness represents intensity and false‐color scale represents lifetime). Each image displayed represents the cumulative sum of all photons detected in a 20 s period, a minimal period which provides sufficient signal for analysis. Over the 30 min duration of the experiment, the time‐resolved spectra, obtained via FLIM, allowed us to identify the increasing Chl intensity as the combination of two signals distinguished by their fluorescence lifetimes. This suggests the presence of two types of membranes, each with their own molecular organization and unique photophysics: i) inside the corrals the predominant signal was a relatively long fluorescent lifetime similar to that observed for the steady state of the washed hybrid membranes (red square features in Figure 4a); ii) across the image globular particles with a short fluorescent lifetime became more numerous over time (large blue/green spots in Figure 4a), presumably representing extracted thylakoids that had not merged with the synthetic lipid bilayers. At later time points (after 300 s), the intensity owing to extracted thylakoids continued to increase, ultimately obscuring the long‐lifetime signal underneath. To quantify the rate of membrane deposition, we generated frequency distribution plots of the fluorescence lifetimes for each 20 s timepoint (Figure 4b). As anticipated from the FLIM images, we observed a bimodal distribution, consisting of a long lifetime peak and a short lifetime peak, consistent with the frequency distribution plots from the steady‐state samples of hybrid membranes and extracted thylakoids (Figure 2j). The lifetime distribution for each timepoint was deconvoluted into two Gaussian populations, as shown in Figure 4c (acceptable fits were achieved for all timepoints, with *R* ^2^ > 0.9). The peak amplitude of each Gaussian, representing the size of the subpopulation, was plotted against time to determine the rate of deposition for each type of membrane (Figure 4d). The amplitude for extracted thylakoids increased with time in a roughly linear manner suggesting a progressively increasing surface coverage (blue line in Figure 4d). This signal may be expected to saturate after a sufficiently long time as the surface becomes completely covered by extracted thylakoids, but the deposition process was stopped before this point was reached. For hybrid membranes, the amplitude increased at a much faster rate reaching a maximum value at ≈500 s (green line in Figure 4d). It is likely that the saturation effect arises from the effect of filling the finite area within the corral regions. This is consistent with the model for a Langmuir isotherm,^[^ ^26^ ^]^ where the rate of material adsorption is proportional to the remaining free space on the substrate. In the early stages of the Langmuir model where there is a large amount of remaining free space, the rate of deposition is almost linear, and then starts to slow down, and eventually saturate, as the surface becomes increasingly occupied.

The introduction of synthetic lipids (DOPC) was essential for the formation of the hybrid membranes, and thylakoids did not merge with the template in the absence of DOPC.^[^ ^17^ ^]^ If a glass surface without a template pattern was used, thylakoids on their own adsorbed as randomly sized aggregates (Figure S7a, Supporting Information). When a mixture of DOPC and thylakoid was incubated on glass, again the thylakoids were found as distinct particles, but now with a continuous lipid bilayer in between (Figure S7b, Supporting Information). Here, the thylakoid particles were not directly connected with the lipid bilayer as shown by lack of lateral protein diffusion via fluorescence recovery after photobleaching (FRAP). Alternatively, if a contiguous DOPC membrane was preformed within a template and thylakoid membrane added afterward, then the thylakoid membranes only loosely associated with the DOPC membrane surface and were washed away with buffer flow (Figure S7c,d, Supporting Information). This demonstrates that it is not merely the presence of DOPC or the template alone that triggers the insertion of LH and PS proteins into the bilayer but the combination of the accessible Diyne‐PC edge and a developing DOPC bilayer. To investigate the role of the synthetic lipids more directly, a small amount of fluorescently tagged lipids were incorporated into DOPC vesicles (0.5% wt/wt Texas Red lipids), before mixing with extracted thylakoids. Time‐lapse FLIM was performed on this sample to track the fluorescence specific to lipids during hybrid membrane formation, and images were analyzed as above (red line in Figure 4d). By fitting each peak amplitiude curve to the Langmuir model (Section S10, Supporting Information, for model derivation), the rate of lipid deposition was found to be 5.5 times greater than the rate of protein deposition (*R*_lipids_ = 0.039, *R*_proteins_ = 0.007). As a result, the lipid amplitude saturates much earlier (*t*_sat_ = 100 s for tagged lipids, *t*_sat_ = 600 s for Chl‐proteins), and FLIM images show the lipid fluorescence was homogeneous across the corral region at *t* = 100 s, suggesting a close to, or completely, fluid DOPC bilayer at this time (Figure S8, Supporting Information). Our results suggest that a bilayer of synthetic lipids is largely assembled inside the corral, before the majority of photosynthetic proteins have been incorporated into the membrane: in fact, the amplitude representing proteins assembling in hybrid membranes increases another two‐ to threefold after the lipid signal has saturated (compare green vs red curve at *t* = 100 s in Figure 4d).

The lack of protein incorporation when thylakoids are deposited on top of a preformed lipid bilayer (Figure S7c,d, Supporting Information) indicates that it is unlikely the proteins are able to transfer vertically between the thylakoid and the DOPC membranes (this would expose hydrophobic portions of the protein to the polar solvent which would be thermodynamically unfavorable). Therefore, it seems probable that proteins and thylakoid lipids migrate laterally between lipid bridges that form between the ruptured thylakoids and patches of putative hybrid membrane. In examples where particularly large extracted thylakoids had adhered on top of a corral, chlorophyll fluorescence appeared to spread from these particles into the surrounding area, see images in Figure 4e. Therefore, we hypothesize that extracted thylakoids adhere on top of the nascent DOPC lipid bilayer and act as reservoirs, from which photosynthetic proteins undergo diffusion down a concentration gradient into the spreading hybrid membrane, as proposed in the cartoon in Figure 4e. Random (Brownian) motion in 2D is expected to lead to an overall migration of membrane proteins from a high concentration in the thylakoids to a lower concentration in the hybrid membranes,^[^ ^27^ ^]^ tending toward a lower energy state of dynamic equilibrium. This interpretation is in agreement with other studies which observed lipid and cofactor diffusion between multilayers of stacked model membranes^[^ ^28^ ^]^ and bears similarities to the dynamic protein rearrangements which occur in natural thyalkoids.^[^ ^7^, ^10^ ^]^

After hybrid membrane formation and stabilization (washing to remove any loosely associated material), FRAP experiments were performed to assess the nature of protein and lipid diffusivity in the hybrid membrane, as shown in Figure 4f. The rate of diffusion was calculated by tracking the fluorescence intensity as a function of time in sequential images in an area of the membrane that was deliberately bleached with intense white light in comparison to a reference area (blue vs red dashed regions, Figure 4f). Our results show that LH and PS proteins have a diffusion rate that is ≈50% slower than lipids in hybrid membranes. Mobile fraction calculations show that the majority of proteins (77%) and lipids (97%) were able to laterally diffuse within the membrane apparently unimpeded by any interactions with the substrate (Figure S9 and Tables S6 and S7, Supporting Information). It is possible that the method of hybrid membrane formation favors the generation of a system with mobile proteins, as only those proteins that are able to diffuse from the adsorbed thylakoids would be incorporated. This process could cause selective sorting of proteins into one transmembrane orientation with the exclusion of proteins with an orientation where bulky extramembraneous protrusions would come into contact with the substrate. For example, PSII protrudes asymmetrically from the lipid bilayer (≈4 nm on the lumenal side, compared to ≈1.7 nm on the stromal side)^[^ ^12b^ ^]^ and is likely to be immobile if the lumenal side experiences friction with the underlying substrate.

### 2.3 Quantitative Analysis of Protein Density Using Atomic Force Microscopy

**Figure 5:**
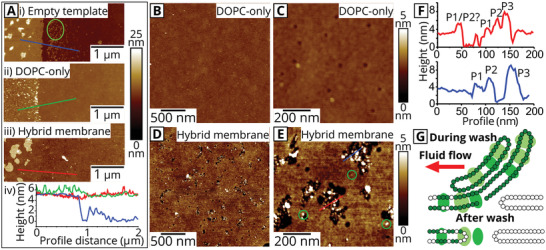
High‐resolution AFM analysis of empty templates versus hybrid membranes. a) AFM image centered on the step from the Diyne‐PC template to the middle the corral for i) an empty template (the green ringed area shows possible effects photolithography pattern blurring), ii) a DOPC‐only lipid membrane and iii) a hybrid membrane. iv) Height profiles across this step edge plotted for each scenario, with lines colored as in (i)–(iii). b) AFM image of an area at the center of a corral backfilled with a DOPC lipid bilayer. c) AFM image of (b) at higher magnification. d) An area at the center of the corral backfilled with hybrid membrane. Pores are visible with this resolution. e) AFM image of (d) at higher magnification where particles are observed within the pores and lipid bilayers (ringed green). f) Height profiles from the red and blue lines shown in panel (e), showing three possible types of immobilized proteins found within pores (denoted P1, P2, and P3). g) A schematic for a possible mechanism for the formation of membrane pores. See Figure S11 (Supporting Information) for a full gallery of AFM topographs.

To identify structural features that are specific to presence of material from the thylakoid membranes, we compared the topography of DOPC‐only lipid membranes formed with the template to the topography of hybrid membranes. Initially, a high‐resolution, stand‐alone AFM was used to confirm the thickness of the hybrid membrane as ≈4.5 nm by direct comparison of the step height at the edge of a corral before and after backfilling (**Figure** **5**a), in agreement with our other measurements. Note that DOPC‐only lipid membranes and hybrid membranes had a similar thickness (green and red lines in Figure 5a(iv)). Further AFM measurements were performed at the center of the corrals to avoid any pattern‐related imperfections at the corral edge that could adversely affect the membrane structure (multiple Diyne‐PC patches can be observed in Figure 5a(i), ringed green, as a result of the resolution limit of the UV patterning). As described in previous sections, multiple small pores were observed in the hybrid membrane (Figure 5d,e); however, these pores were not present in membranes formed solely from DOPC. AFM measurements showed a contiguous and defect‐free DOPC‐only membrane (Figure 5b,c) confirming the quality of our synthetic lipid vesicles and suggesting that the small breaks within the hybrid membranes are because of the presence of thylakoid membranes. At the base of many of the pores, and embedded within the membrane (Figure 5e, ringed green), static particles were observed. These particles typically range from 5 to 10 nm in height (height profiles in Figure 5f) and could be classified into subpopulations that roughly agree with the dimensions expected for LH and PS proteins^[^ ^12b^ ^]^ (P1–P3 in Figure 5f). These particles may therefore represent a variety of photosynthetic proteins which have become immobilized at these locations (further statistical analysis of these populations is shown in Figure S10, Supporting Information). One possibility is that these pores form when thylakoids that are loosely associated or have partially fused with the SLB stripped away from the surface, pulling away sections of the membrane and leaving some material stuck to the substrate (shown schematically in the cartoon in Figure 5g).

Qualitatively, significantly fewer particles can be seen in the DOPC‐only membranes (Figure 5b,c) than in hybrid membranes (Figure 5d,e). To estimate the percentage of particles that represent thylakoid membrane proteins, a careful manual analysis was performed to compare the particle density in hybrid membranes to the particle density in lipid‐only membranes. Protrusions where counted as “protein‐candidate” particles if they were between 5 and 10 nm in height and ≈10–20 nm in diameter, as expected for the thylakoid membrane proteins.^[^ ^12b^ ^]^ Our analysis showed that the particle density was significantly higher for hybrid membranes (77.0 particles µm^−2^) than for the control sample of DOPC‐only (9.7 particles µm^−2^). This suggests that ≈80% of the particles observed in hybrid membranes by AFM can be confidently identified as LH or PS proteins, leading to an estimated protein density of ≈60 proteins µm^−2^. This approximated protein density from AFM measurements was on the same order, but lower than our previous estimates of protein density calculated via fluorescence intensity (estimations of ≈60 proteins µm^−2^ from AFM compared to 80–450 proteins µm^−2^ from fluorescence). This difference can be explained by considering that only static proteins can be observed by AFM and that any highly mobile molecules would be “invisible” to the slow raster speed of the AFM probe. From our FRAP calculations of mobile fraction, discussed earlier, we hypothesize that the protein density estimated by AFM only represents a small minority (≈20%) of the total population. After taking the “invisible” mobile population into account, our approximated protein density from AFM comes into good agreement to the approximated protein density calculated via fluorescence (AFM estimate of ≈300 proteins µm^−2^, FLIM estimate of between 80 and 450 proteins µm^−2^).

### 2.4 Assessing Photosynthetic Activity: Model Membranes Highlight Potential Flaws in Commonly Used Functionality Assays

**Figure 6:**
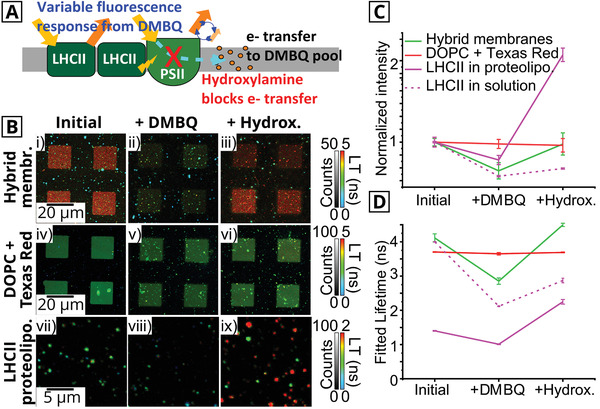
Quantification of possible photochemical activity using hybrid membranes and FLIM. a) A schematic showing the possibilities for photon absorption, energy transfer, and electron transfer in a hybrid membrane (various biomolecules after PSII not shown for simplicity). After DMBQ addition, excitation energy will drive electron transport from PSII to DMBQ, instead of producing Chl fluorescence. After the addition of hydroxylamine, the PSII is no longer able to perform photochemistry, and electron transfer is blocked, resulting in increased Chl fluorescence to dissipate the excess excitation energy. b) FLIM measurements of hybrid membranes, and DOPC/Texas Red lipid membranes and LHCII proteoliposomes, before and after the addition of 0.5 × 10^−3^ m DMBQ and, after the addition of 0.5 × 10^−3^ m hydroxylamine, as labeled. The FLIM instrument was set up with 640 nm excitation and 672–696 nm emission for (i)–(iii) and (vii)–(ix), or with 561 nm excitation and 590–650 nm emission for (iv)–(vi). Spectral overlap between these FLIM channels was minimal, see Table S2 (Supporting Information). c) Graph showing the normalized fluorescence counts of each sample plotted against the different experimental conditions from (b) (averaged over four fields of view). d) Graph showing the mean fluorescence lifetimes for each sample against the experimental conditions from (b) (averaged over four fields of view). The raw data for the cuvette‐based spectroscopy of LHCII proteoliposomes and LHCII in detergent solution are shown in Figure S12 (Supporting Information).

Finally, we attempted to assess the photosynthetic activity of the hybrid membranes, specifically, the transduction of excitation energy into electron transport (photochemistry). If this functionality is even partly retained in hybrid membranes, then this model system could be used to investigate these fundamental processes, or, owing to the ability of PSII to donate electrons to downstream inorganic systems, may have applications in future photoelectronic technologies.^[^ ^29^ ^]^ Multiple studies have proposed that, by selectively switching on or off portions of the electron transfer energy process, various photochemical inhibitors can give an indirect measure of the activity of PSII.^[^ ^30^ ^]^ Therefore, we performed a “photochemical assay” on hybrid membranes by monitoring changes to the Chl fluorescence intensity (and lifetime), in response to these inhibitors. In hybrid membranes as prepared, the water‐soluble proteins responsible for electron transport from PSII to other proteins are likely to be missing (**Figure** **6**a). In this scenario, absorbed energy from PSII would be primarily released as Chl fluorescence. In the first stage of the assay to test the system, an exogenous electron acceptor, DMBQ, is introduced to the membranes at a relatively high concentration, to replace the natural electron carriers (PQ) which are likely to be saturated.^[^ ^31^ ^]^ If DMBQ successfully accepts electrons from PSII, the level of Chl fluorescence should be reduced in its presence compared to its absence, because excitation energy can be used to eject electrons rather than being reemitted. In the final stage of the assay, “hydroxylamine” can be added as an aqueous solution and is known to increase the Chl fluorescence again (Figure 6a).^[^ ^32^ ^]^ Hydroxylamine is a small highly reactive compound, reported to affect various cofactors within PSII, disrupting the oxygen‐evolving complex and inhibiting the electron transfer cycle.^[^ ^33^ ^]^ The photochemical assay described above was performed on hybrid membranes and control samples and characterized by FLIM. Figure 6b(i) and (ii) show that for hybrid membranes the Chl fluorescence intensity is indeed significantly quenched after the addition of DMBQ, to 55% (±13%) of its original intensity. The additional decay mechanism resulted in the decreased fluorescence lifetime within the corrals of hybrid membrane (from red to green on the false‐color FLIM scale). Upon the addition of hydroxylamine, the fluorescence intensity recovered back to 97% (±17%) of its initial intensity (Figure 6b(iii)). The trends for fluorescence intensity and for fluorescence lifetime of hybrid membranes are shown as green lines in the graphs of Figure 6c,d, respectively. The fluorescence lifetime was reduced from 4.11 ± 0.12 ns initially to 2.85 ± 0.09 ns upon the addition of DMBQ, before recovering to 4.49 ± 0.05 ns upon the addition of the hydroxylamine. This final lifetime is longer than in the initial system and could be owing to changes in the configuration of pigments within PSII after the addition of hydroxylamine.

A series of control samples were studied to assess the specificity of this assay on photosynthetic proteins and for probing electron transfer. DOPC lipid membranes containing the fluorophore Texas Red (without any proteins) showed typical images of patterned membranes (Figure 6b(iv)–(vi)), with no significant change at any stage of the photochemical assay of either the fluorescence lifetime or the fluorescence emission intensity. This showed that DMBQ does not cause quenching of this chromophore, and hydroxylamine does not affect it either (red lines in Figure 6c,d). Photochemical assays were then performed on control samples of both LHCII proteins within membranes that were deposited onto glass (solid magenta lines in Figure 6c,d) versus and isolated LHCII proteins suspended in an aqueous solution (dashed magenta lines in Figure 6c,d). These were assessed as samples which contained Chl but not PSII and represented the quenched or nonquenched states of LHCII, respectively. Therefore, these samples are examples of photosynthetic proteins that lack the electron transfer functionality inherent in PSII that this assay is expected to probe. Surprisingly, the DMBQ caused quenching of the fluorescence intensity and lifetime in both LHCII‐only samples, followed by the subsequent dequenching by hydroxylamine. LHCII in proteoliposomes showed significantly less DMBQ‐induced quenching and more hydroxylamine‐induced dequenching compared to LHCII in solution, presumably because LHCII in proteoliposomes started in an already heavily quenched state. These positive results for LHCII suggest that the DMBQ/hydroxylamine assay is not specific for detecting electron transfer, as LHCII does not normally release electrons. A direct “collisional quenching” mechanism where DMBQ interacts with Chl excited states to cause deexcitation seems possible (either by diffusing through the lipid bilayer or through the aqueous solution).^[^ ^34^ ^]^ Hydroxylamine must also lack specificity for binding to and disrupting PSII, suggesting that this chemical could have the potential to react with and disrupt any Chl‐containing protein. The increased Chl fluorescence lifetime of >4 ns suggests that hydroxylamine may either cause some sort of disaggregation of Chls which increases the intraprotein Chl—Chl distance, or a chemical change to the Chls which decreases the dipole–dipole coupling. While we cannot definitively rule out the possibility that the glass surface alters the structure or chemical activity of LH or PS protein complexes, we note that previous studies have concluded that LHCII and other protein complexes are not adversely affected by interactions with glass^[^ ^13^ ^]^ or mica^[^ ^9d^ ^]^ surfaces (comparisons of their fluorescence emission spectra indicate that the native protein conformation is maintained).

These photochemical experiments demonstrate one major advantage of using a range of model membrane systems which are assembled from the desired components, namely, that we can perform measurements on membranes containing only one type of protein to illuminate potential issues. Our comparisons of LHCII in a quenched and nonquenched state also demonstrate that the initial photophysical state of the proteins can significantly alter the outcomes or interpretations of functionality assays. A virtue of the use of FLIM (over intensity measurements) is that we can quantify such photophysical states more definitively to give confidence in our interpretations. Despite the apparent lack of specificity of the photochemical assays, they show that our hybrid membranes contain “active” Chl which responds to chemical modifications in a very different manner to other fluorophores, such as Texas Red.

## 3 Conclusions

Hybrid membranes have distinct advantages when compared to native thylakoids or other model systems as a platform to study the fundamentals of photosynthesis. Natural thylakoid extracts are heterogeneous, micro/nanoscale structures, and are difficult to study owing to their relative instability. Alternative models, while more robust, are typically formed by “bottom‐up” approaches that use purified proteins and lipids to exert control over composition, at the expense of missing many important components and a resulting simplification of the complex interactions observed in natural membranes.^[^ ^9^, ^14^, ^15^, ^20^ ^]^ Hybrid membranes offer an intermediate situation and consist of a stable, flat, and largely homogeneous structure (observed by FLIM and AFM) formed via self‐assembly from natural membranes, such as all the natural LH and PS proteins are potentially available for analysis. Our FLIM and FRAP measurements show that the proteins are highly mobile (≈80% mobile fraction) and are free to interact with the surrounding lipids/proteins in a way that is unimpeded by the substrate. The protein concentration (≈1% of the membrane area) is significantly lower than that of native membranes; however, there are many possible avenues which could be pursued in future studies to increase the protein concentration of the hybrid membranes to better represent the native system, such as altering the concentrations of the starting material^[^ ^17^ ^]^ or by using techniques established in the SLB community to direct the diffusion of membrane proteins.^[^ ^35^ ^]^ The combination of FLIM and AFM allowed us to observe the dynamic behaviors and interactions of individual elements of photosynthesis (i.e., LH and PS protein complexes) in a controllable platform and opens the possibility to manipulate them. The fluorescence lifetime increased from ≈0.5 ns in natural membranes to ≈4 ns in the hybrid membranes, which suggests that the chlorophyll‐proteins are switching from a quenched to a light‐harvesting state as the protein concentration decreases. Such changes in fluorescence lifetime have been previously suggested to relate to energy‐dissipating pathways within LHCII being switched on and off and the crucial process of “photoprotection” in plants.^[^ ^9^ ^]^ It seems feasible that future studies could explore the fluorescence switching of single LH and PS proteins by using a hybrid membrane platform. Another key advantage of using model membrane systems is that specific proteins of interest can be investigated, as shown in our application of our hybrid membranes and proteoliposomes to photochemical assays. This revealed new challenges in accurately determining electron transport using DMBQ and hydroxylamine, suggesting that the assays used in the photosynthesis community may have to be reassessed.^[^ ^32^, ^33^ ^]^

By imaging the self‐assembly of lipids and photosynthetic proteins onto the solid surface in real time, we found that both the hydrophobic edge of the Diyne‐PC corral and the developing DOPC bilayer are necessary for the formation of flat and contiguous membranes (possibly owing to free energy or energy minimization considerations^[^ ^36^ ^]^) from the highly curved natural membranes. This suggests that the polymerized lipid template could be used to support the formation of SLBs from a range of biological membranes that are otherwise difficult to study (high‐curvature, protein‐dense). In future, it may also be possible to take advantage of the self‐assembly mechanism of hybrid membrane formation to introduce additional lipophilic components of interest (e.g., additional pigments for light‐harvesting)^[^ ^14f^ ^]^ or to generate desirable membrane architecture that might mimic the stacked structure of chloroplasts. Previous studies have shown that it is possible to generate self‐assembling multilayered lipid membranes by exploiting electrostatic attractions between anionic lipids and cationic polymers,^[^ ^28^, ^37^ ^]^ divalent cations,^[^ ^38^ ^]^ or protein–protein interactions (including LHCII–LHCII).^[^ ^14b^ ^]^ It may be possible to modify our methodology in similar ways to generate multilayered Diyne‐PC templates to address the stacked nature of bioenergetic membranes.

## 4 Experimental Section

### Preparation of Extracted Thylakoids, Purified LHCII, and Lipid/Protein Vesicles

Thylakoid membranes were isolated from spinach (*Spinacia oleracea*) as described by Morigaki and co‐workers.^[^ ^17^ ^]^ Briefly, this involved macerating leaves at 4 °C, disruption of the chloroplasts by passing them through a high‐pressure vessel and recovery of thylakoid membranes in an aqueous buffer (50 × 10^−3^ m KH_2_PO_4_, 10 × 10^−3^ m NaCl, 2 × 10^−3^ m MgCl_2_, 330 × 10^−3^ m sorbitol, pH 7.5). Absorption spectroscopy confirmed that the membranes contained the expected optically active proteins (LHCII, PSII, PSI, see Figure S1, Supporting Information). These “extracted thylakoids” were used to form hyrbid membranes within a few days or were flash‐frozen with liquid nitrogen and stored at −80 °C. Purified trimeric LHCII (required for control measurements) was extracted and purified from spinach leaves following established procedures using detergent (*n*‐dodecyl‐alpha‐d‐maltopyranoside), sucrose density gradient sedimentation, and size‐exclusion chromatography (purity was confirmed by denaturing and native gel electrophoresis).^[^9d^]^ LHCII proteoliposomes were prepared by combining specified quantifies of lipids, detergent, and purified LHCII protein in an aqueous buffer and then inducing the self‐assembly of membranes by removing the detergent using Biobeads, as described previously.^[^ ^14f^ ^]^ Lipid vesicles, required for hybrid membranes formations, were formed from high‐purity DOPC lipids following standard probe sonication procedures, with Texas Red lipids (Texas Red 1,2‐dihexadecanoyl‐*sn*‐glycero‐3‐phosphoethanolamine) included only when tracking lipids were required.

### Preparation of Polymerized Lipid Templates and Hybrid Membranes

The polymerized lipid templates were prepared as described in several previous publications.^[^ ^18a,b^ ^]^ Briefly, lipid bilayers of 1,2‐bis(10,12‐tricosadiynoyl)‐*sn*‐glycero‐3‐phosphocholine (Diyne‐PC) were deposited onto substrates by vesicle spreading and then polymerization was conducted by UV irradiation using a mercury lamp, using very careful control over power delivered, process temperature, and presence of oxygen. Substrates patterned with polymerized Diyne‐PC could be stored in water for weeks at room temperature. Immediately before use, patterned substrates were dried with nitrogen and placed into a microscopy sample holder as desired (either ultrathin adhesive imaging spacers to confine the sample droplet or the AFM original equipment manufacturer coverslip holders). Extracted thylakoids and DOPC vesicle suspensions were combined in a 1:3 wt/wt ratio and added to the substrate at a final concentration of 0.68 × 10^−3^ m DOPC. After 30 min incubation, samples were rinsed with copious buffer solution and were ready for microscopy.

### Atomic Force Microscopy

Standalone AFM was performed under aqueous buffers using a Bruker Dimension FastScan and PEAKFORCE‐HIRS‐SSB probes (Bruker AFM Probes) in PeakForce tapping mode. Parameters were optimized while imaging to minimize applied forces of <0.2 nN, typically scanning at 2–4 Hz and 1024 × 1024 pixels. Topographs were processed and analyzed using Nanoscope Analysis Software (v1.9). For combined FLIM+AFM, the AFM imaging used a JPK NanoWizard 4 driven by a Vortis Advanced control station. A JPK Tip Assisted Optics stage was used for a sample‐scanning configuration, so that once the FLIM laser spot and AFM probe were aligned they remained in a fixed position to ensure consistent correlation between the two systems and minimal noise.

### Fluorescence Microscopy

FLIM was performed using a Microtime 200 time‐resolved fluorescence microscope (PicoQuant GmbH). This system used an Olympus IX73 inverted optical microscope as a sample holder with light passing into and exiting various filter units for laser scanning, emission detection, and timing electronics. Excitation lasers (picosecond pulsed sources) were driven in pulsed interleaved excitation mode by a PDL 828 Sepia II burst generator module. The pulse width for the LDH 485 nm, LDH 561 nm, and LDH 640 nm lasers were 90, 70, and 90 ps, respectively. Detector 1 was a single‐photon avalanche diode and detector 2 was a hybrid photomultiplier tube. Specific dichroic mirrors and emission filters, as described in the text, were used to define the emission channel wavelength range. An excitation fluence of 0.012 mJ cm^−2^ was used which allowed sufficient fluorescence signal while limiting any singlet–singlet annihilation events (optimization shown in Figure S13 and Tables S8 and S9, Supporting Information). Similarly, we ruled out any significant effects owing to the presence or absence of dissolved oxygen in the buffer on the photophysical properties of the proteins (Figure S14, Supporting Information). Analysis of all FLIM data was performed with SymPhoTime software (PicoQuant). The mean amplitude‐weighted lifetime of images or specific objects, 〈τ〉, was calculated by generating fluorescence decay curves from accumulated photons, and then modeling the curve as a multiexponential decay function (excellent fits were achieved for all data, with chi‐squared values <1.1 and low residuals).

## Conflict of Interest

The authors declare no conflict of interest.

## Supporting information

Supporting Information

Supplemental Video 1

Supplemental Video 2

## Data Availability

All raw data and analysis associated with this paper are openly available under a CC‐BY license in the Research Data Leeds repository^[^ ^39^ ^]^ under https://doi.org/10.5518/884.
