# Supplementary material for: Model Lipid Membranes Assembled from Natural Plant Thylakoids into 2D Microarray Patterns as a Platform to Assess the Organization and Photophysics of Light‐Harvesting Proteins
Source: Small. 2021 Mar 9;17(14):2006608. doi: 10.1002/smll.202006608 (PMC11476343; doi:10.1002/smll.202006608)
Supplement: Supplementary file 1 — Supporting Information [file SMLL-17-2006608-s001.pdf]

© 2021 Wiley-VCH GmbH

NANO MICRO small

Supporting Information

for *Small,* DOI: 10.1002/smll.202006608

Model Lipid Membranes Assembled from Natural Plant Thylakoids into 2D Microarray Patterns as a Platform to Assess the Organization and Photophysics of Light-Harvesting Proteins

Sophie A. Meredith, Takuro Yoneda, Ashley M. Hancock, Simon D. Connell, Stephen D. Evans, Kenichi Morigaki, and Peter G. Adams**

© 2021 Wiley-VCH GmbH

Supporting Information

Model Lipid Membranes Assembled from Natural Plant Thylakoids into 2-D Microarray Patterns as a Platform to Assess the Organization and Photophysics of Light-Harvesting Proteins

Sophie A. Meredith, Takuro Yoneda, Ashley M. Hancock, Simon D. Connell, Stephen D. Evans, Kenichi Morigaki, and Peter G. Adams**

S. A. Meredith, Dr. A. M. Hancock, Dr. S. D. Connell, Prof. S. D. Evans, Dr. P. G. Adams
School of Physics and Astronomy and Astbury Centre for Structural Molecular Biology, University of Leeds, Leeds, LS2 9JT, United Kingdom
E-mail: p.g.adams@leeds.ac.uk

T. Yoneda, Dr. K. Morigaki,
Graduate School of Agricultural Science and Biosignal Research Center, Kobe University, Rokkodaicho 1-1, Nada, Kobe, 657-8501, Japan
E-mail: morigaki@port.kobe-u.ac.jp

1. Ensemble absorption and fluorescence spectroscopy of extracted thylakoids show they contain all the expected photosynthetic components

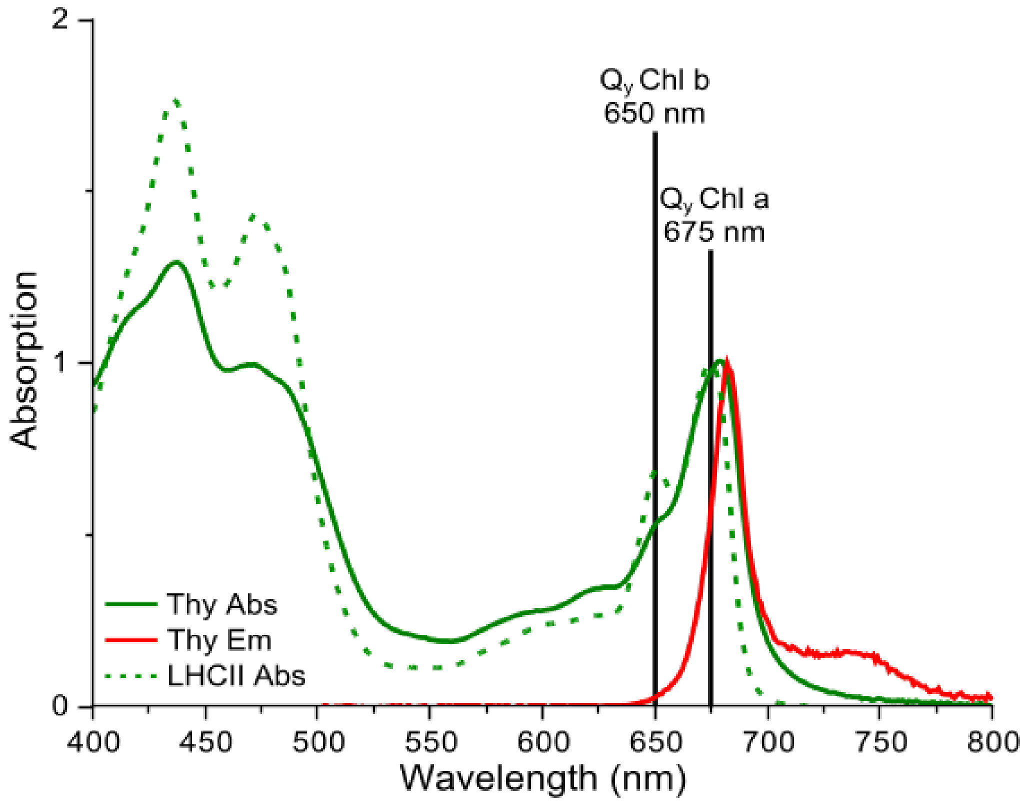

Figure S1: Emission (*solid red*) and absorption (*solid green*) data for extracted thylakoids and absorption data for LHCII (*dashed green*). Cuvette-based absorption spectroscopy was performed using an Agilent Technologies Cary 5000 UV-Vis-NIR absorption spectrophotometer. Cuvette-based fluorescence spectroscopy was performed using an Edinburgh Instruments FLS980 system, using excitation at 473 nm and collecting emission from 500-800 nm (2 nm and 1 nm bandwidth excitation and emission slits, respectively). The absorption spectra from both samples share some features, with similar peaks but at subtly different maximum wavelengths and with varying peak intensities. As expected, the overlapping peaks due to carotenoids and the chlorophyll Soret bands are found between 400-500 nm and the known Q_y bands for chlorophylls at 650-700 nm.

Taking a closer look at the LHCII absorption spectrum (*green dashed line*), there is a clear peak at precisely 675 nm and a lower intensity peak at precisely 650 nm, representing Chl *a* and Chl *b* Q_y transitions, respectively. The ratio of peak heights between Chl *a* and Chl *b* is as expected for LHCII and the subtle shoulder at 475 nm is indicative of the trimeric form of LHCII. These peak wavelengths and intensities are in excellent agreement with previous reports of the optical properties of spinach LHCII.[1] Gel electrophoresis of the purified protein shows the expected bands, as previously reported by our group.[1a, 1b]

In comparison, the extracted thylakoids absorption spectrum (*solid green*) is more complex, with contributions from LHCII, PSII and PSI. There is a peak centred at ~680 nm which is significantly broader than for LHCII and there is only a shoulder at ~650 nm, rather than a distinct peak. In summary, this spectrum is very similar to published reports for extracted thylakoids.[2] In detail, the well-established features of plant thylakoids, are explained by the following: (i) PSI and its antenna has maximal Chl *a* absorption at ~682 nm as compared to the PSII at a maximum of ~677 nm, explaining why this peak is found at longer wavelength compared to isolated LHCII alone, (ii) the PSI peak is known to extend further into the red due to its more numerous low-energy chlorophylls, explaining the observed broadening of the peak towards the red end of the spectrum, (iii) furthermore, PSII has much lesser Chl *b* than LHCII, and PSI has even less again, explaining the reduced peak at ~650 nm. This is in good agreement with reports by Caffarri and co-workers[2] and others.

The extracted thylakoids fluorescence emission spectrum (*solid red*) has one major peak at ~682 nm and broad "red tail" between 700-750 nm represents emission from low energy Chl *a* (and its extended vibrionic manifold)[3] indicating that the highly connected chlorophyll network across photosynthetic proteins has been maintained in the preparation of the "extracted thylakoids" sample, again, as we would have expected.

2. Quantification of spectral overlap between targeted FLIM channels (confirming minimal nonspecific fluorescence)

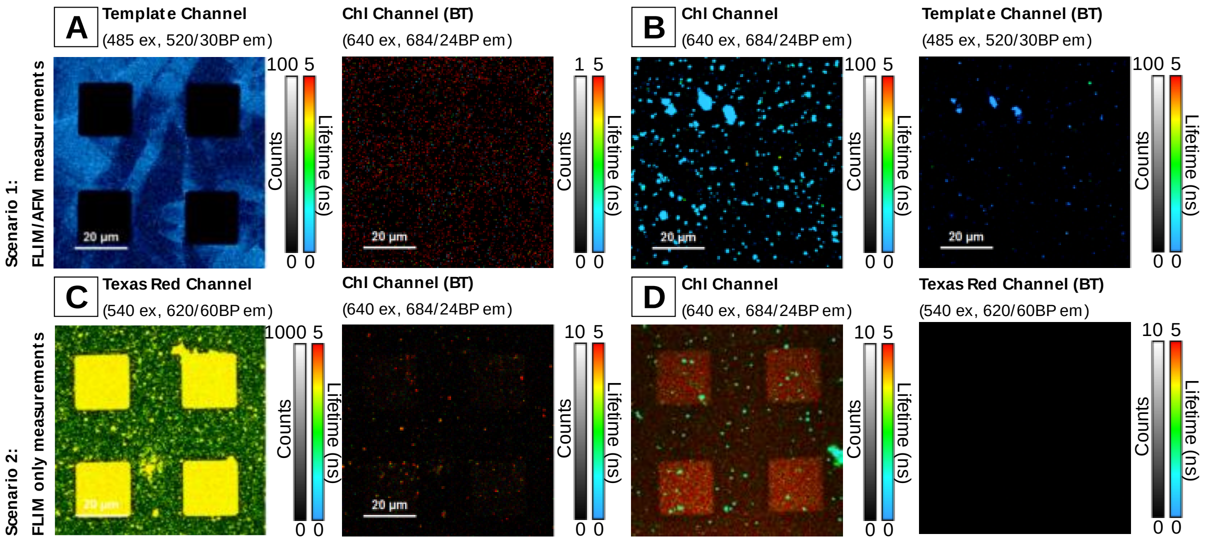

Figure S2: Examples of the spectral crossover between FLIM channel for two scenarios. In the first scenario, for correlated FLIM+AFM measurements, we quantify the spillover between the intrinsic polymerized lipid fluorescence and chlorophyll-related channels. **(A)** FLIM image of a polymerized lipid sample (photo-patterned Diyne-PC), as observed in the "Template channel" (excited at 485 nm, emission at 520 nm with a bandpass width of 30 nm), and the spillover (BT) into the "Chl channel" (excitation at 640 nm, emission at 684nm with a bandpass width of 24 nm). The spectral spillover of the polymerized lipid emission to the Chl channel is less than 1%. **(B)** FLIM image of a sample containing non-ruptured thylakoid extracts, as observed in the Chl channel and the Template channel. The chlorophyll pigments can be excited in their Soret band, which overlaps significantly (2.78%) with the Template channel. However, since the polymerized lipid is acting as a pattern scaffolding, and not the focus of this study, we believe that this overlap into the Template channel is acceptable. In the second scenario, FLIM only measurements are optimized for the detection of Chl fluorescence and Texas Red fluorescence. **(C)** FLIM image of a patterned lipid bilayer composed of 99.5% DOPC + 0.5% TR DHPE in the "Texas Red channel" (excitation at 540 nm, emission at 620 nm with a bandpass width of 60 nm), and the Chl channel (optics as above). The spectral spillover from the TR emission to the Chl channel is less than 1% **(D)** FLIM image of hybrid thylakoid membranes (containing no TR) as seen in both the Chl channel, and spillover into the Texas Red channel. The spectral overlap from the chlorophyll emission into the Texas Red channel is 10.5 ± 1.7 %, however since the TR signal is typically 50-100 times higher than the Chl signal, the overall contribution is negligible.

FLIM measurements were acquired in two scenarios, for which the fluorescence spillover was quantified between the fluorescence channels of interest.

Firstly, correlated FLIM-AFM measurements were used to probe the topography of thylakoid/DOPC hybrid membranes as they backfilled into template polymerized lipid corrals (Figures 2, 3 and 4 in the main text). In order to be confident about the detected fluorescence

signal, we quantified the fluorescence spillover on samples containing polymerized lipids only (“empty” scaffold patterns) and extracted thylakoids only (extracted thylakoids adhered to glass). Multiple fields of view were acquired per sample, using the same laser settings (pulse rate, intensity, etc) as would be used for subsequent measurements. The average number of fluorescence counts for the control samples are shown below in Table S1, where the spill over between the two channels has been calculated. In each case, the spill over between the two fluorescent channels was found to be negligible.

**Sample**	**Template channel** (counts)	**Chl channel** (counts)	**Spill over to Template** (%)	**Spill over to Chl** (%)
Diyne-PC template	$(3.58 \pm 0.46) \times 10^6$	$(5.09 \pm 1.96) \times 10^3$	N/A* (100)	0.12 ± 0.01
Isolated thylakoids	$(1.22 \pm 0.57) \times 10^6$	$(4.41 \pm 0.68) \times 10^7$	2.78 ± 0.42	N/A* (100)

Table S1: Mean counts from control samples in order to calculate the extent of channel spill over from the polymerized lipid channel (excited at 485nm, emission detected at 610-630 nm) to the Chl FLIM channel (excited at 640 nm, emission detected from 672-696 nm) and vice versa. The asterisks (*) denote that this is 100% by definition.

In the second scenario, standalone FLIM measurements were used to quantify spill over when the fluorophore TR was introduce into the sample to track lipid deposition. In this scenario, TR was excited using a 560 nm laser pulse, and detected in the range from 590-650 nm (TR channel). The Chl fluorescence was excited using a 640 nm laser pulse and detected in the range from 672 – 696 nm (Chl channel). FLIM measurements of hybrid membranes (No TR) and TR-only (TR-DOPC) control samples show that there is minimal overlap from Chl emission into the TR channel and vice versa. Table S2, below, shows the mean number of counts inside the corrals (N = 16 corrals) for various samples in each FLIM channel. For the hybrid membranes sample (Table S2, row 1), the crossover from the Chl fluorescence to the TR channel was calculated to be 10.49 ± 1.69 %. However, number of counts in the TR channel is approximately 40-fold higher than that in the Chl channel, therefore the Chl crossover contributes less than half a percent of the total counts in the TR channel and is negligible. From the DOPC-TR sample (Table S2, row 2), the crossover from the TR to the Chl channels was calculated to be 0.07 ± 0.02 %, Therefore in a hybrid membranes sample also containing TR, after considering the relative magnitudes of the TR and Chl signals, the crossover from the TR fluorescence is expected to account for ~3% of the total counts in the Chl channel. In either case, the crossover between Chl and TR channels is sufficiently small, that we can be confident in the fluorescence lifetimes measured in these channels.

Sample	Chl channel (counts)	TR channel (counts)	Spill over to Chl (%)	Spill over to TR (%)
(a) hybrid membranes	(1.08 ± 0.08) × 10^6	(1.14 ± 0.13) × 10^5	N/A* (100)	10.49 ± 1.69
(b) lipid membranes (DOPC +Texas Red)	(3.09 ± 0.57) × 10^4	(4.41 ± 0.68) × 10^7	0.07 ± 0.02	N/A* (100)

Table S2: Mean counts observed inside the corrals (N=16 corrals) of either hybrid membranes (thylakoids/DOPC) or lipid membranes containing 0.5% TR lipids. The extent of fluorescence spill over is calculated for both channels. The asterisks (*) denote that this is 100% by definition.

3. Example FLIM images of empty polymerized lipid templates

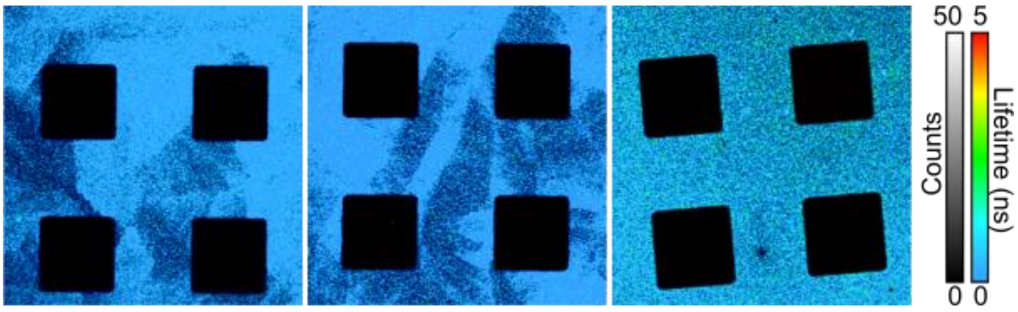

Figure S3: Multiple FLIM images of empty polymerised templates. Diyne-PC SLBs were polymerized by exposure to UV light through a photomask. Non-polymerized regions are then washed away by detergents, resulting in a patterned SLB with empty spaces and high-energy exposed bilayer edges.

4. Hybrid membranes are structurally consistent across multiple preparations – FLIM gallery and homogeneity analysis

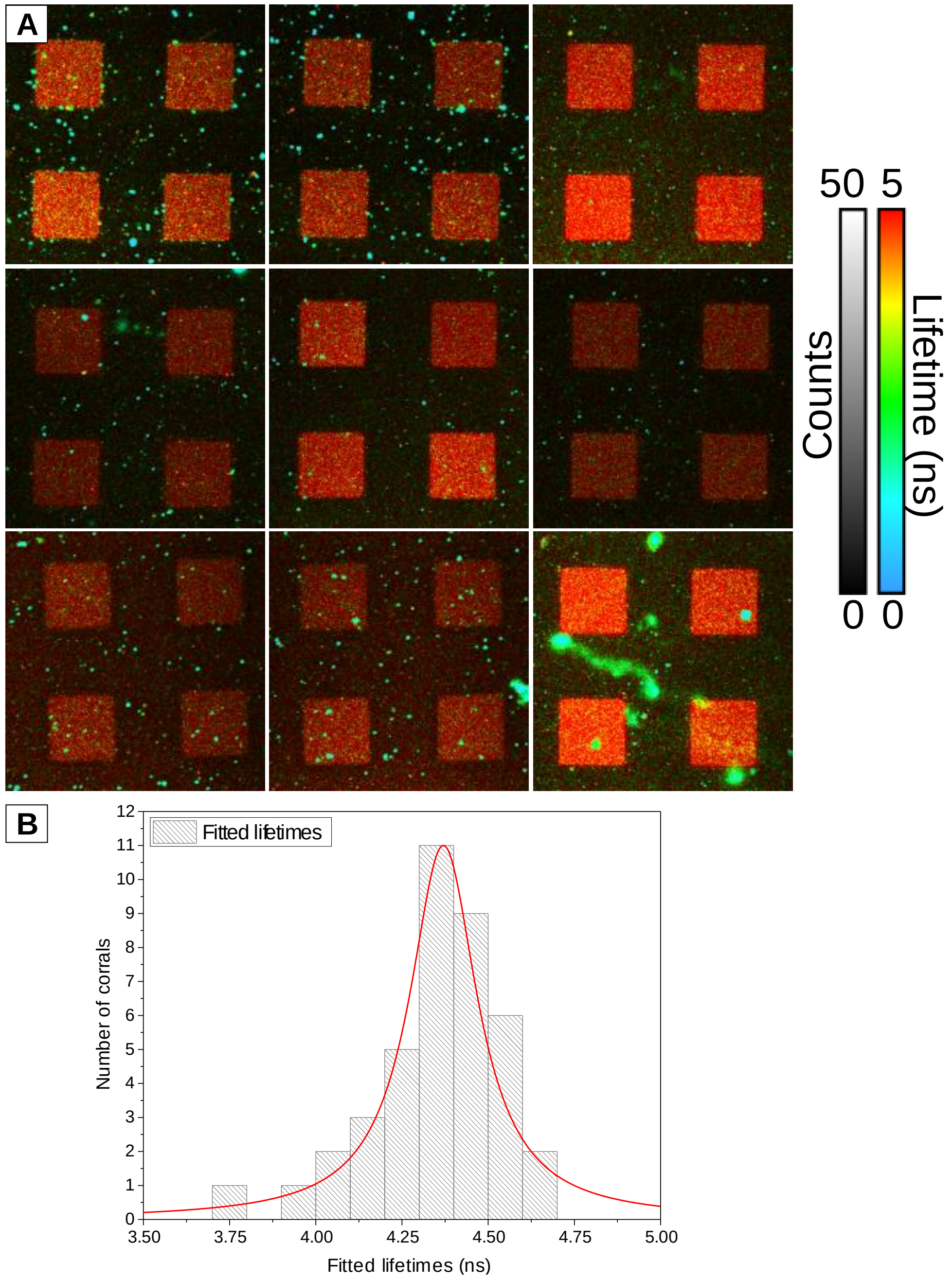

Figure S4: (A) Multiple FLIM images of hybrid membranes within polymerized Diyne-PC templates. Samples were prepared across multiple different days and are seen to be highly reproducible in terms of their array patterned structure. The fluorescence intensity observed did vary between corrals (some are clearly brighter than others) but this could be due to a degree of photobleaching or other effects. Importantly fluorescence lifetime is similar seen here as a similar red color. **(B)** A histogram of fitted fluorescence lifetimes accumulated from 40 individual corrals. The fluorescence lifetime is very similar between hybrid membrane samples with a narrow distribution of Chl lifetimes (FWHM = 0.12 ns). This suggests the protein-protein interactions within the membrane are similar across multiple preparations.

5. FLIM gallery of LHCII proteoliposomes adhered onto glass and calculations from these images to estimate the average fluorescence intensity per proteoliposome

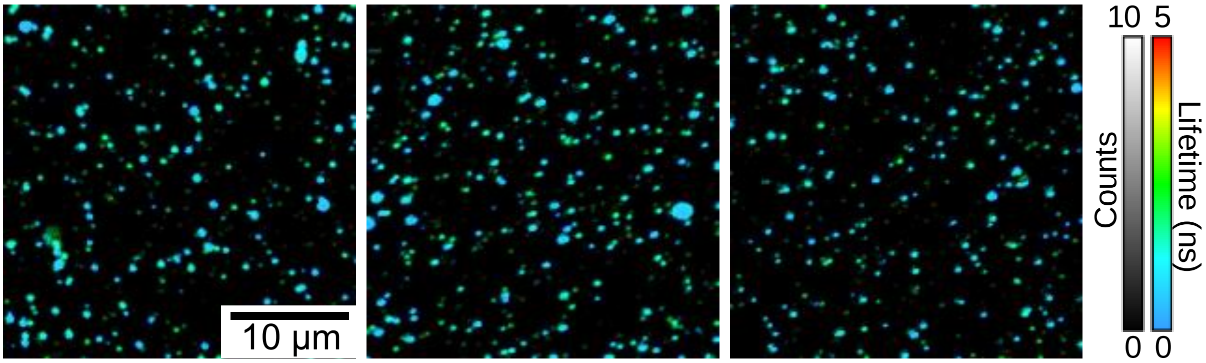

Figure S5: Multiple fields of view of LHCII proteoliposomes (containing approximately 2.8 μM LHCII and 1 mM thylakoid lipids) adhered onto cleaned glass. The samples were imaged in a buffer containing 40 mM NaCl, 20 mM, HEPES, pH 7.5 using the same acquisition settings and laser fluence used for the hybrid membranes, giving a direct comparison of the fluorescence intensity of a known amount of LHCII.

**Proteo-liposome number**	**Frames**	**Noise per pixel per frame** (counts)	**Number of selected pixels** (pix)	**Total counts** (counts)	**Detector noise in the selected area** (counts)	**Corrected $F_{vesicle}$** (counts)
1	500	3.68×10^{-4}	42	155	7.73	147.27
2	500	3.68×10^{-4}	32	17	5.89	11.11
3	500	3.68×10^{-4}	21	71	3.86	67.14
4	500	3.68×10^{-4}	39	30	7.18	22.82
5	500	3.68×10^{-4}	30	47	5.52	41.48
6	500	3.68×10^{-4}	20	71	3.68	67.32
7	500	3.68×10^{-4}	23	126	4.23	121.77
8	500	3.68×10^{-4}	34	182	6.26	175.74
9	500	3.68×10^{-4}	63	18	11.59	6.41
10	500	3.68×10^{-4}	57	152	10.49	141.51

Table S3: Calculation of the fluorescence intesnity signal for 10 example LHCII proteoliposomes (from images as shown in Figure S5). This "corrected" signal represents the number of counts (photons emitted) per proteoliposome after subtracting the detector noise. For a population of N=100 proteoliposomes, the average fluorescence intensity was found to be 74.58 ± 7.66 counts for a 500-frame acquisition. This equates to an **average $F_{vesicle}$** of 0.149 counts per frame.

6. Calculations for the average number of proteins within typical LHCII proteoliposomes

Scenario	D (nm)	L/P (mol/mol)	$A_{vesicle}$ (nm^2)	A_{lipid} (nm^2)	A_{LHCII} (nm^2)	n (LHCII /vesicle)	density (LHCII /μm^2)
best estimate	60	1071	11310	0.5	50.3	35.5	3143
min. estimate	100	1071	31416	0.45	38.5	21.0	2680
max. estimate	50	1071	7854	0.55	78.5	112.4	3577

Table S4: Calculations for the number of LHCII proteins found on average per proteoliposome, given the estimated dimensions for the protein, lipids and the vesicle. This is for the proteoliposome sample shown in Figure S5 and Table S3 (which has 2.8 μM LHCII and 1 mM thylakoid lipids).

D, average diameter measured via dynamic light scatterting measurements (DLS), 60 nm is the average but given the accuracy of DLS the low and high values shown represent reasonable low and high estimates;

L/P, the average lipid-to-LHCII trimer ratio, as determinated from ensemble absorption spectroscopy measurements and spectral decomposition analysis using published methodology;

$\mathbf{A_{vesicle}}$, calculated from $4\pi r^2$ (where, r= D/2);

$\mathbf{A_{lipid}}$, published value for DOPC headgroup area,[4] given the uncertainty we use 0.45 and 0.55 as the low and high estimates;

$\mathbf{A_{LHCII}}$, estimation of the membrane area occupied by one LHCII, from the consideration of space-filling models of published protein structures[4-5] and then approximation of LHCII as a circular area (πr^2) where r = 3.5, 4.0 or 5.0 for the low, medium and high estimates (range due to uncertain protein packing);

Area per vesicle approximates to the following equation (note, the factor 0.5 is due to 2 lipids one from each two monolayer togethe to form one bilayer and thus occuping an area of A_{lipid}):

$$A_{vesicle} = n[A_{LHCII} + 0.5(L/P)A_{lipid}]$$

This expression was solved to calculate **n** using the values for **L/P**, $\mathbf{A_{vesicle}}$, $\mathbf{A_{lipid}}$ and $\mathbf{A_{LHCII}}$.

Density = n / $A_{vesicle}$.

The minimum, maximum and best estimates are made using the different possible values shown for each term, as shown.

7. Calculations for the average number of proteins within a corralled hybrid membrane

Scenario	$N_{LHCII/vesicle}$ (#)	$F_{vesicle}$ (counts/frame)	$\tau_{vesicle}$ (ns)	F_{LHCII} (counts/frame)	F_{corral} (counts/frame)	$N_{LHCII/corral}$ (#)	Density (LHCII/µm^2)	A_{LHCII} (nm^2)	$A_{protein}$ (%)
best estimate	35.5	0.149	0.637	0.0263	1503	57100	143	50.3	0.72
min. estimate	21.0	0.149	0.637	0.0446	1503	33700	84	38.5	0.32
max. estimate	112.4	0.149	0.637	0.0083	1503	180000	451	78.5	3.54

Table S5 : Calculations for the number of proteins per corral, in terms of "LHCII-equivalents". This uses the average fluorescence counts measured by FLIM for an LHCII proteoliposome and converts to counts per LHCII protein, given the measured number of proteins within a typical proteoliposome (from Table S4). Consistent acquisition parameters were used to record FLIM images of both LHCII proteoliposomes and hybrid membranes.

$\mathbf{N_{LHCII/vesicle}}$, estimated number of LHCII-equivalents per proteoliposome (**n** from Table S4). This range from the minimum to the maximum considering our combined uncertainties;

$\mathbf{F_{vesicle}}$, estimated fluorescence intensity measured per proteoliposome per frame ("average $F_{vesicle}$" as stated in the caption of Table S3);

$\boldsymbol{\tau_{vesicle}}$, the measured mean fluorescence lifetime of a typical LHCII proteoliposme (mean of N=100 measured particles from images similar to Figure S5);

$\mathbf{F_{LHCII}}$, the FLIM counts expected per LHCII per frame calculated for each possible $\mathbf{N_{LHCII/vesicle}}$, as follows. LHCII within proteoliposomes is known to self-quench, shortening the fluorescence lifetime due to the self-association of neighbouring LHCII.[1b] The measured $\tau_{vesicle}$ of proteoliposomes of 0.637 ns (SD = 0.015 ns) implies significant quenching relative to isolated LHCII in detergent ($\tau_{DDM} \approx$ 4ns), so to crudely take this into account we can multiply by the ratio of the lifetimes (4/0.637). Thus, the intensity of the proteins in the unquenched state is estimated as:

$$\mathbf{F_{LHCII}} = \frac{\mathbf{F_{vesicle}}}{\mathbf{N}_{\left(\frac{\mathbf{LHCII}}{\mathbf{vesicle}}\right)}} \times \left(\frac{\tau_{DDM}}{\tau_{vesicle}}\right) = \frac{0.149}{\mathbf{N}_{\left(\frac{\mathbf{LHCII}}{\mathbf{vesicle}}\right)}} \times \left(\frac{4}{0.637}\right) = \frac{0.94}{\mathbf{N}_{\left(\frac{\mathbf{LHCII}}{\mathbf{vesicle}}\right)}}$$

$\mathbf{F_{corral}}$, average fluorescence intensity measured in FLIM of hybrid membranes, as total counts within one corral per frame. This vale is found from careful analysis of the corrals from many images of hybrid membranes similar to those shown in Figure S4 (N = 16 corrals);

$\mathbf{N_{LHCII/corral}}$, is the estimated number of LHCII trimers per corral, N = F_{corral} / F_{LHCII};

Density (LHCII/corral) = $\mathbf{N_{LHCII/corral}}$ / A_{corral} (where each corral has area A_{corral} = 400 µm^2);

$\mathbf{A_{LHCII}}$, is the area occupied by a single trimeric LHCII protein complex, as estimated in Table S4;

$\mathbf{A_{protein}(\%)}$, estimated surface area fraction of the corral occupied by LHC and PS proteins:

$$A_{protein}(\%) = Density(\mu m^2) \times A_{LHCII}(nm^2)/10^6$$

8. Further data: AFM gallery of thylakoid membranes

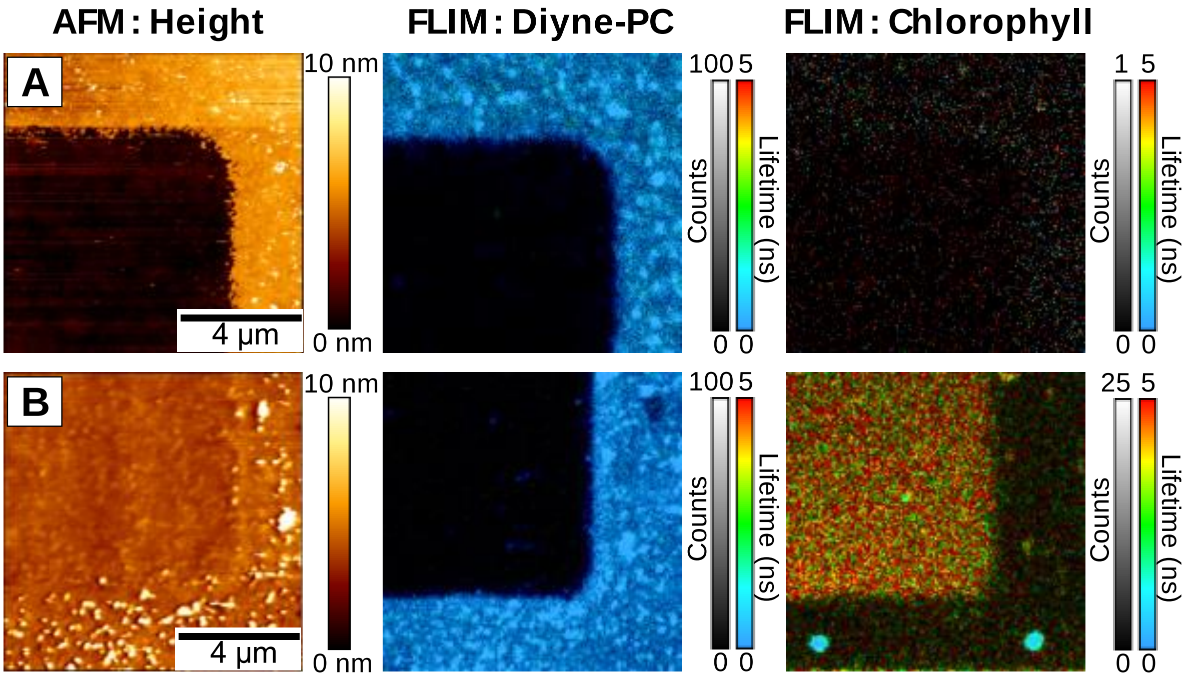

Figure S6: Additional correlated FLIM/AFM measurements at a higher magnification than those shown in the main text. For (A+B), left panel: AFM topograph; center panel: "polymer FLIM channel", i.e., optimized to detect the polymerized lipid fluorescence (laser excitation at 485 nm, emission bandpass between 505-535 nm); right panel: "chlorophyll FLIM channel", i.e., optimized to detect the fluorescence of chlorophylls which is expected from thylakoid LHC and PS proteins (laser excitation at 640 nm, emission bandpass between 672-696nm). **(A)** Correlated FLIM+AFM data showing a single square of the polymerized lipid "empty" template. The minimal signal in the chlorophyll FLIM channel is statistically indistinguishable from detector noise. **(B)** Correlated FLIM+AFM data showing a similar region as in (A), but after it was backfilled with the extracted thylakoids and liposomes to form the hybrid membrane within the corrals.

9. Control measurements showing the requirements for high-quality Hybrid Membranes: thylakoids only merge with DOPC membrane if they are mixed in presence of the diyne-PC template

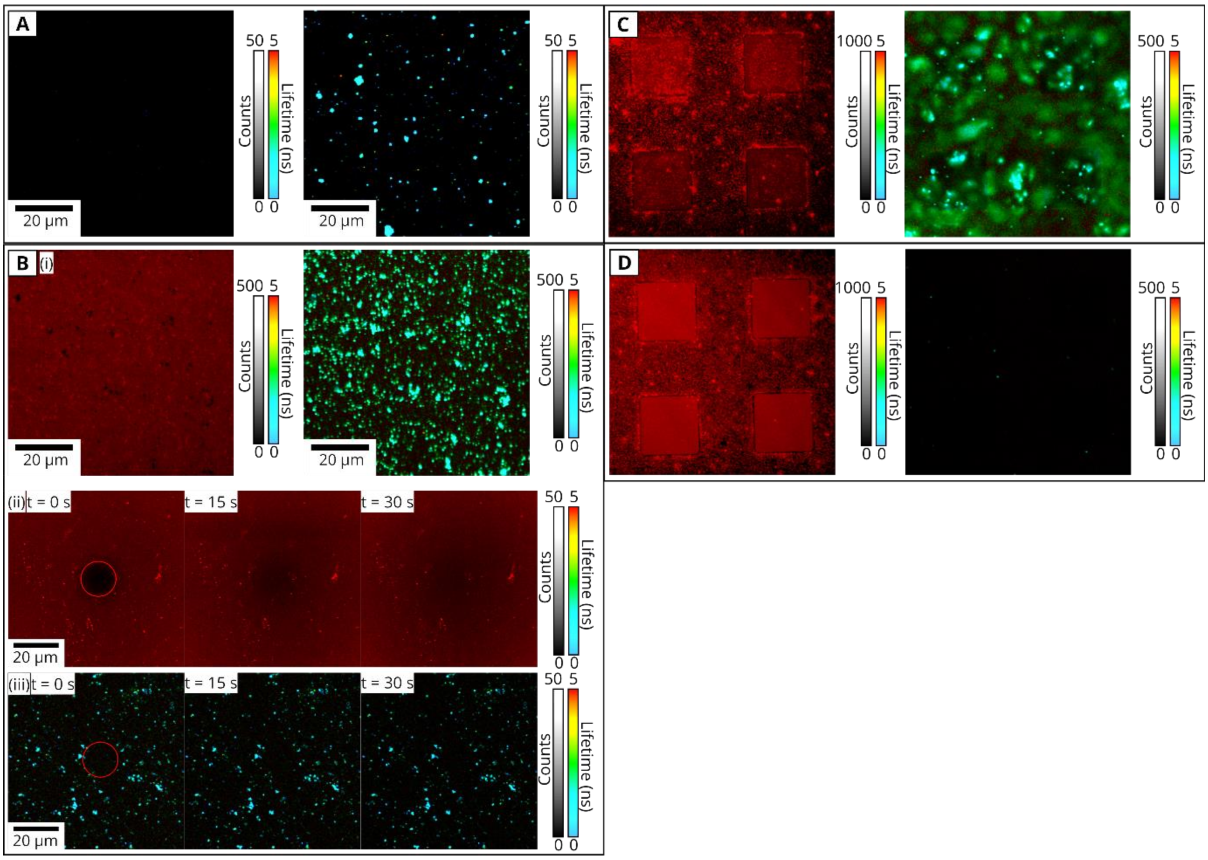

Figure S7: The deposition of thylakoid extracts onto glass substrates in different conditions to investigate what determines the formation of high-quality membranes. *Left column:* fluorescence due to lipids (Texas Red DHPE). *Right:* Fluorescence due to the thylakoid proteins (chlorophyll). **(A)** FLIM image of thylakoid extracts deposited on a glass surface. The sample was prepared by incubating the standard concentration of thylakoid extracts (0.18 mg/mL) with a hydrophilic (piranha-cleaned) glass coverslip for 30 min. The sample was washed with copious buffer solution before imaging. **(B) (i)** FLIM image of a mixed assembly of thylakoid membranes and DOPC SLBs on glass. The sample was prepared by mixing the standard 1:3 ratio (weight/weight) of extracted thylakoid membranes with DOPC vesicles as an aqueous suspension, and then depositing these onto a hydrophilic glass coverslip for 30 minutes. The sample was washed with copious buffer solution before imaging. Here, the DOPC liposomes were labelled with 0.5% (mol/mol) Texas Red-DHPE. **(ii), (iii)** Photobleaching measurements to test the lateral diffusion of the lipids and proteins shown in **(i). (C)** FLIM image after thylakoid extracts were incubated with "pre-formed" lipid membranes within the standard Diyne-PC template pattern, before any washing procedures. This sample was prepared in a two-stage process: (i) a template pattern was incubated with 1 mg/mL DOPC vesicles labelled with 0.5% (mol/mol) Texas Red-DHPE for 30 min and then washed with buffer solution, (ii) this surface was then incubated with the standard concentration of thylakoid extracts. Microscopy was performed immediately after this. **(D)** FLIM image after washing the sample from (C) with copious buffer. As can be observed, no thylakoid membranes remained. All images are acquired with the same FLIM parameters as FLIM figures in the main text. The standard aqueous buffers described in the main text were used for all.

Figure S7A shows that thylakoids membranes will adhere to hydrophilic glass surfaces and remain as distinct particles (in the absence of DOPC or any template). This is a similar sample to main text Figure 2A and the data is in agreement.

Figure S7B shows that if a glass surface without a template was used, the thylakoid membranes did not appear to merge with the DOPC membrane and appeared to remain as distinct particles, as shown from the Chl fluorescence (*right panel*). Whereas, the DOPC formed a sort of patchy supporting lipid bilayer (*left panel*) around the thylakoid particles. The lack of connectivity of the thylakoid membrane is shown from the lack of protein mobility beyond these particles in the FRAP data in **Figure S7Biii** (i.e., lack of recovery of fluorescence after photobleaching), whereas the lipids within the synthetic membrane showed some degree of lateral diffusion in **Figure 27Bii**, suggesting that they formed as usual (i.e., recovery of fluorescence after photobleaching). This is evidence that the Diyne-PC template is absolutely required in order for thylakoid membranes to merge with DOPC lipid membrane during the formation process of hybrid membranes.

Figure S7C and **S7D** shows that if a DOPC membrane was pre-formed within a template and thylakoid membrane added afterwards, then the thylakoid membranes only loosely associated with the DOPC membrane surface and were washed away with buffer flow. Note whilst the lipid fluorescence is high (*left panel*), the Chl fluorescence is low (*right panel*) suggesting that minimal thylakoid proteins remained. In Figure S7C, there appears to be no particular pattern of preferential adherence of thylakoid particles to either the template grid or DOPC box region, although this could be obscured by the many out-of-focus particles which are in solution above the surface. This suggests that there are only loose interactions of thylakoids if the lipid bilayer is already complete, as confirmed by the subsequent images acquired after washing the surface with fresh buffer solution.

The fluorescence lifetimes of the Chl fluorescence in **all four scenarios** is very short (~0.5 ns) and similar to the lifetime of thylakoid extracts. This suggests that the density of proteins is still very high, and that proteins have not been able to outwardly diffuse from the thylakoids into a lower-density hybrid membrane which has a longer fluorescence lifetime (~4 ns).

All together, this is evidence that the thylakoid membranes must mix with the spreading edge of nascent forming DOPC lipids. LH and PS protein do not transfer vertically from thylakoid membranes into pre-existing DOPC supported lipid bilayers.

10. Derivation of the Langmuir isotherm model

In the Langmuir isotherm model, the rate of material deposition is proportional to the amount of remaining free space on the surface. This assumes that each absorption site is energetically identical.[6]

This can be expressed mathematically as $\frac{dn}{dt} = R\left(1 - \frac{n}{N}\right)$, where n is the number of absorped molecules, R is a constant that describes the rate of absorption, and N is the total number of possible sites.

To solve this differential equation, we can consider two possible scenarios. In the first scenario, $n \ll N$, and there are a large number of remaining empty sites. The surface is not close to saturation.

In this scenario,

$$\frac{dn}{dt} = R\left(1 - \frac{n}{N}\right) \approx R$$

and therefore, $n = Rt + C$, where C is a constant arising from the integration of $\frac{dn}{dt}$. The rate of deposition is initially linear. This scenario was applied to fit the deposition of extracted thylakoids, where the surface coverage was very low throughout the incubation period.

In the second scenario, $n \sim N$, and the rate of deposition slows down as the number of free sites decreases.

$$\frac{dn}{dt} = R\left(1 - \frac{n}{N}\right)$$

which is rearranged to,

$$\frac{1}{1 - \frac{n}{N}}\, dn = R\, dt$$

This can be integrated to

$$-Nlog(N - n) = Rt + C$$

and rearranged in to the final form, $n = N - e^{\left(-\frac{R}{N}t + C\right)}$, used to fit the data from both the hybrid membrane and DOPC-TR deposition curves.

11. A comparison of lipid deposition rate to protein deposition rate in a sample containing fluorescently-tagged lipids

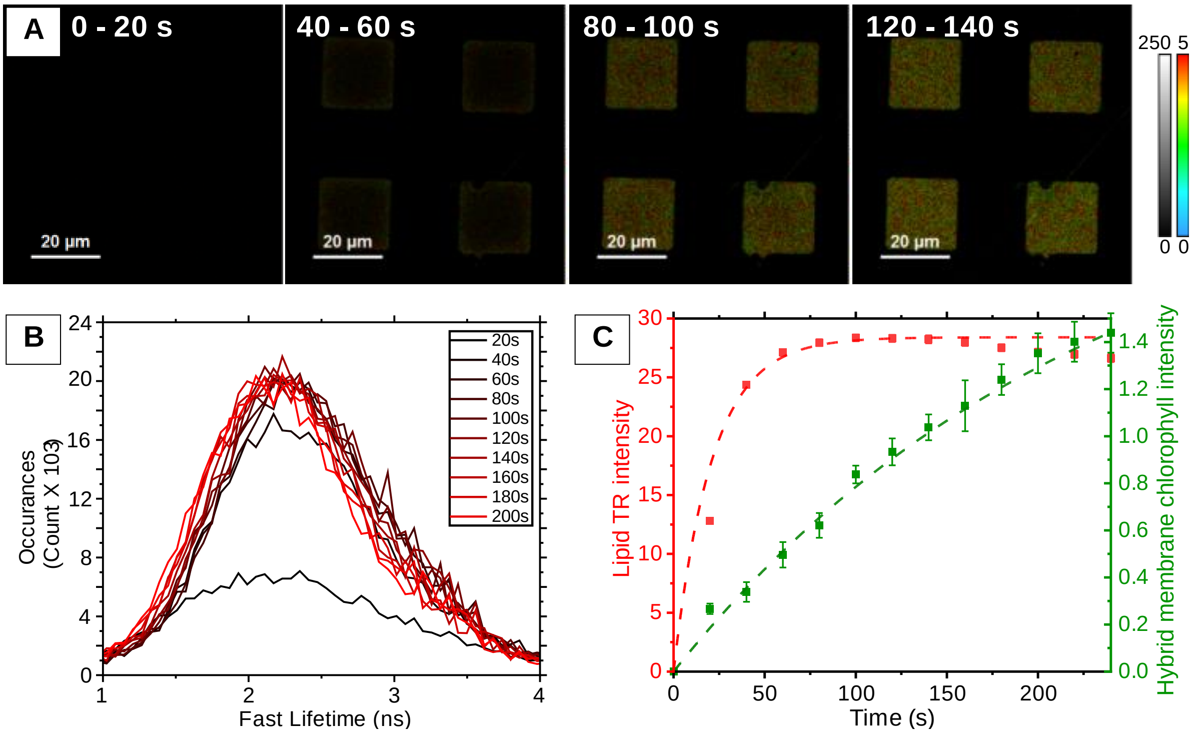

Figure S8: Analysis of the dynamic process of hybrid membrane formation. **(A)** A series of FLIM images showing the deposition of a hybrid membrane containing both photosynthetic proteins and fluorescently tagged (TR-DHPE) lipids. The FLIM channel shown here is optimized for the detection of Texas Red, in order to compare the rate of lipid deposition to the deposition of photosynthetic proteins (excitation at 561 nm and emission collected between 590-650 nm). **(B)** Evolution of the frequency distribution of lifetimes during the deposition. Dark red to light red represents the cumulative frequency distribution at increasing time points of 20-40s, 40-60s, 60-80s, 80-100s, 100-120s, 120-140s, 140-160s, 160-180s, 180-200s. Each distribution is fitted to a Gaussian curve, in order to calculate the Peak amplitude at each point. **(C)** Analysis of the growth over time of the TR intensity (from the peak amplitude data from panel B), compared to the hybrid membrane Chl intensity (from the long-lifetime Chl peak amplitude data from main text Figure 3). The lipid intensity saturates a lot sooner (~60s) than the hybrid membrane Chl intensity (~500s).

12. Fluorescence Recovery After Photobleaching (FRAP) measurements to assess lipid and protein mobility in hybrid membranes

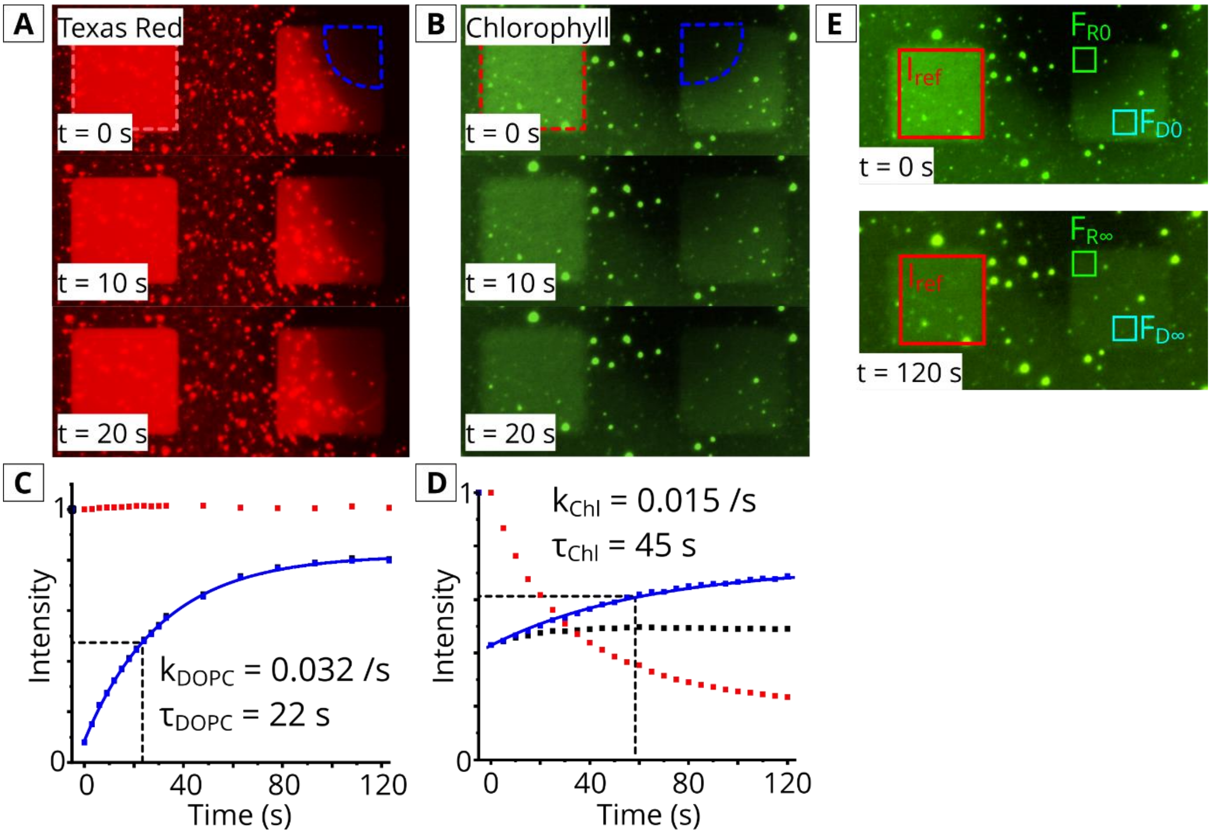

Figure S9: Epifluorescence microscopy with photobleaching (FRAP) experiments to assess the lateral mobility of proteins and lipids within hybrid membranes. Images were acquired at the time-points as labelled on panels. **(A)** An epifluorescence time-lapse image series of a patterned DOPC lipid membrane which contained 1% Texas Red DHPE fluorescently-labelled lipids (mol/mol). The Texas Red fluorophores were bleached within the *blue dashed* region at t = 0 s. The fluorescence can be seen to recover in subsequent images. **(B)** An epifluorescence time-lapse image series of a hybrid membrane. LH and PS proteins were bleached within the *blue dashed* region at t = 0 s, and non-bleached proteins can be seen to diffuse into the bleached region over the subsequent images. **(C)** Analysis of the recovery of fluorescence from (A) representing the lipid diffusion rate. The *red* line shows the fluorescence intensity tracked in a reference corral to correct for any photobleaching that occurs during image acquisition. The *blue* line shows the fluorescence intensity in the bleached area, corrected for photobleaching, as it increases over 120 s. As there is minimal bleaching during imaging the raw data (*black*) and the corrected (*blue*) datapoints overlap. A mono-exponential growth function was fitted to the recovery data (*blue line*), $F(t) = F_0\, e^{kt}$, where t=time, k= rate constant, $F(t)$ is the intensity at time t, F_0 is the initial intensity. This yields a fluorescence doubling-time τ = 22 s ($\tau = ln2/\,k$). **(D)** Analysis of the recovery of fluorescence from (B) representing the photosynthetic protein diffusion rate. This analysis was performed as described for (C) and yielded a doubling-time τ = 45 s. (**E**) A hybrid membrane imaged at t = 0 s, immediately after photobleaching, and at a timepoint significantly after this, t = 120 s. Highlighted areas: I_{ref} (*red)*, a reference area used to track photobleaching. F_R (*green*), the bleached area. F_D (*cyan*) an area in the corral that was not bleached.

To approximate the diffusion constant of LH and PS proteins. FRAP measurements were performed on DOPC (labelled with 0.5% Texas Red (mol/mol) and hybrid membranes deposited into the Diyne-PC templates. The epifluorescence instrument was a Nikon E600 microscope equipped with an Andor Zyla 4.2 sCMOS detector, set up with a filter cube optimized for Chl in for panel A (excitation 450-475 nm, dichroic 500 nm, emission 650-800 nm) and with a filter cube optimized for Texas Red in panel B (excitation 540-580 nm, dichroic 595 nm, emission 600-660 nm). Images were acquired using an x100 oil objective, 0.5s exposure and appropriate ND filters. For deliberate photo-bleaching, an aperture was inserted to expose circular region of ~15 µm diameter of the sample for a continuously period of 30 s at full power (i.e. no ND filters). Immediately following bleaching, full-field images were acquired sequentially over a period of 120 s, and the fluorescence recovery plotted over time.

To account for the rapid photobleaching of the Chl fluorescence, the intensity of an adjacent "reference corral" was also measured at each timepoint. The intensity of the bleached area was then adjusted for this bleaching to provide an estimate for the rate of recovery. A mono-exponential fit, $F = F_0(1 - e^{-kt})$, was used to obtain the "doubling time", $\tau = \ln(2)/k$, for both DOPC and Chl fluorescence, this was found to be 21.5 ± 1.6 s and 44.7 ± 10.3 s respectively (fit ± standard error,). From the relative doubling time, we approximate the diffusion constant of LH and PS proteins to be ~48 % of the diffusion constant of DOPC lipids ($D_{Chl}/D_{DOPC} = \tau_{Chl}/\tau_{DOPC}$).

In addition to the diffusion constant, we attempted to calculate the mobile fraction of the PS and LH proteins bty devising a simple analysis procedure. This uses the logic that the "bleached" region must recover to an intensity equal to the "non-bleached" regions if where the mobile fraction is 100%. **Figure S9E** shows an FRAP experiment where images are taken at t = 0s and t = 120s.

In the scenario where photobleaching is performed in one region within an enclosed reservoir of lipids, there are a finite number of fluorophores that can diffuse into the bleached area (due to the confined area). The mobile fraction is calculated by:

$$M = \frac{F_R}{F_D}$$

Where F_R is the fluorescence intensity of the bleached area, and F_D is the fluorescence of an area within the corral that was not bleached, after the system has been allowed to reach an equilibrium. As F_R recovers, the intensity of F_D decreases as bright fluorophore migrate into the bleached area, and vice versa. In the scenario where there is 100% mobility, $F_R/F_D = 1$ as the fluorescence of the two areas will tend to the same intensity over time.

To account for fluorophores that are not completely bleached after 30 s, the equation takes the form:

$$M = \frac{F_{R\infty} - F_{R0}}{F_{D\infty} - F_{R0}}$$

Where F_{R0} and $F_{R\infty}$ is the intensity of the bleached area at t = 0s and at t = ∞ respectively. In our analysis, we approximate $t = \infty \sim 120\, s$. Both F_R and F_D are corrected for any photo-bleaching that has occurred by tracking the intensity of an adjacent reference corral.

Reference corral (t = 0s) I_{Ref0}	Reference corral (t = 120s) $I_{Ref\infty}$	Photo-bleaching (P)	Bleached area (t = 0s) F_{R0}	Non-bleached area (t = 0s) F_{D0}	Bleached area (t = 120s) $F_{R\infty}$	Corrected for photobleaching $F_{R\infty}/P$	Non-bleached area (t = 120s) $F_{D\infty}$	Corrected for photobleaching $F_{D\infty}/P$	Mobile fraction M
458.32	131.97	0.29	60.52	327.51	90.72	315.05	110.16	382.56	0.79
332.80	81.45	0.24	38.16	127.16	56.22	229.74	78.22	319.63	0.68
383.23	89.63	0.23	35.30	169.84	64.69	276.59	74.72	319.50	0.85
381.07	89.64	0.24	47.01	193.17	70.66	300.38	86.67	368.44	0.79
286.30	84.90	0.30	34.12	132.27	52.62	177.43	75.02	252.97	0.65
286.30	84.90	0.30	37.51	125.44	49.48	166.87	56.51	190.55	0.85

Table S6 : Calculations for mobile fraction of LH and PS proteins in hybrid membranes.

$\mathbf{I_{Ref0}}$ and $\mathbf{I_{Ref\infty}}$, the fluorescence intensity of a reference corral, measured at time t = 0 s and t = 120 s respectively. This is used to calculate the extent of photobleaching, **P**, that occurs during the FRAP acquisition as: $\mathbf{P = 1 - I_{Ref\infty}/I_{Ref0}}$.

$\mathbf{F_{R0}}$ and $\mathbf{F_{R\infty}}$, the fluorescence intensity of the bleached area, measured at t = 0 s and t = 120 s respectively.

$\mathbf{F_{D0}}$ and $\mathbf{F_{D\infty}}$, the fluorescence intensity of a non-bleached area in the same corral as the bleached area, measured at t = 0 s and t = 120 s respectively.

M, the mobile fraction calculated from equation described above. The average mobile fraction across multiple measurements was calculated to be 76.8 ± 3.1 % (mean ± standard error, N = 6).

To verify this method of calculating the mobile fraction, we also calculated the mobile fraction for lipids (labelled with 0.5% Texas Red DHPE). We expect the mobile fraction for lipids to be close to 100%. Using the calculations shown in Table S7, we find the mobile fraction to be 96.9 ± 0.7 % (mean ± standard error, N = 6).

Reference corral (t = 0s) I_{Ref0}	Reference corral (t = 120s) $I_{Ref\infty}$	Photo-bleaching (P)	Bleached area (t = 0s) F_{R0}	Non-bleached area (t = 0s) F_{D0}	Bleached area (t = 120s) $F_{R\infty}$	Corrected for photobleaching $F_{R\infty}/P$	Non-bleached area (t = 120s) $F_{D\infty}$	Corrected for photobleaching $F_{D\infty}/P$	Mobile fraction M
414.49	405.56	0.98	67.86	482.40	223.86	228.79	228.40	233.43	0.97
414.49	405.56	0.98	89.77	507.67	262.14	267.91	265.86	271.72	0.98
426.93	426.84	1.00	26.53	428.75	175.92	175.96	183.57	183.61	0.95
426.93	426.84	1.00	17.56	334.15	112.72	112.74	118.99	119.01	0.94
426.93	426.84	1.00	76.02	437.44	327.69	327.76	332.69	332.76	0.98
426.93	426.84	1.00	59.81	422.35	282.34	282.40	283.82	283.88	0.99

Table S7 : Calculations for mobile fraction of lipids in hybrid membranes.

$\mathbf{I_{Ref0}}$ and $\mathbf{I_{Ref\infty}}$, the fluorescence intensity of a reference corral, measured at time t = 0 s and t = 120 s respectively. This is used to calculate the extent of photobleaching, **P**, that occurs during the FRAP acquisition as: $\mathbf{P = 1 - I_{Ref\infty}/I_{Ref0}}$.

$\mathbf{F_{R0}}$ and $\mathbf{F_{R\infty}}$, the fluorescence intensity of the bleached area, measured at t = 0 s and t = 120 s respectively.

$\mathbf{F_{D0}}$ and $\mathbf{F_{D\infty}}$, the fluorescence intensity of a non-bleached area in the same corral as the bleached area, measured at t = 0 s and t = 120 s respectively.

M, the mobile fraction calculated from equation described above.

13. Classification of protein protrusion within defects in the bilayer

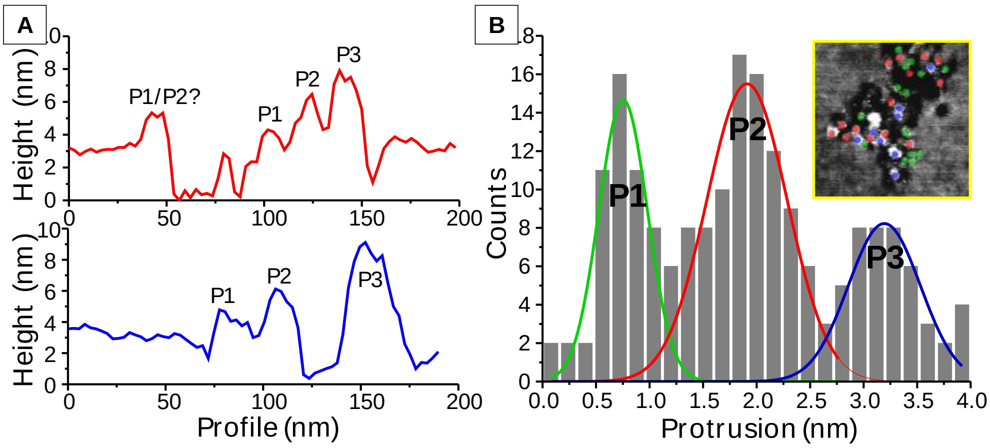

Figure S10: Quantitative analysis of particle heights within a thylakoid hybrid membrane. **(A)** AFM height traces of particles within the defects, seem to reveal 3 distinct types of particles (labelled P1, P2 and P3). **(B)** A particle height histogram obtained from profile analysis across N=250 particles. Height traces were drawn across individual particles, as in (A), and the difference in particle height from the surrounding bilayer (protrusion) across many particles was accumulated as a histogram. Within this population, 3 peaks are observed with corresponding particle protrusions of 0.753 ± 0.027 nm (assigned P1), 1.911 ± 0.035 nm (assigned P2) and 3.194 ± 0.062 nm (assigned P3) respectively. The inset shows an example region, where particles have been color coded (P1 = *green*, P2 = *red*, P3 = *blue*) corresponding to their particle classification.

To classify the proteins, we used the much higher (~0.1 nm) z axis resolution of the AFM. Statistical analysis of the profiles across hundreds of proteins (N = 250) reveals a protrusion (i.e., the maximum height above the lipid bilayer) distribution with three distinct populations (see Figure 3I). These populations can be fitted to a Gaussian distribution ($R^2 > 0.99$), and were found to protrude from the bilayer by 0.753 ± 0.027 nm (assigned P1), 1.911 ± 0.035 nm (assigned P2) and 3.194 ± 0.062 nm (assigned P3) respectively. Our results are in moderate agreement with the known crystal structures of LHCII, PSII and Cytb$_6$f, and with previous AFM studies that have used the same approach.[8] The P1 peak is consistent with the predicted height for LHCII, and the P2 and P3 peaks show the same relative heights for PSII and Cytb$_6$f, respectively. From this we conclude that hybrid membranes contain many of the relevant photosynthetic membrane proteins.

14. Further data: gallery of AFM images of hybrid membranes observed at a range of magnifications

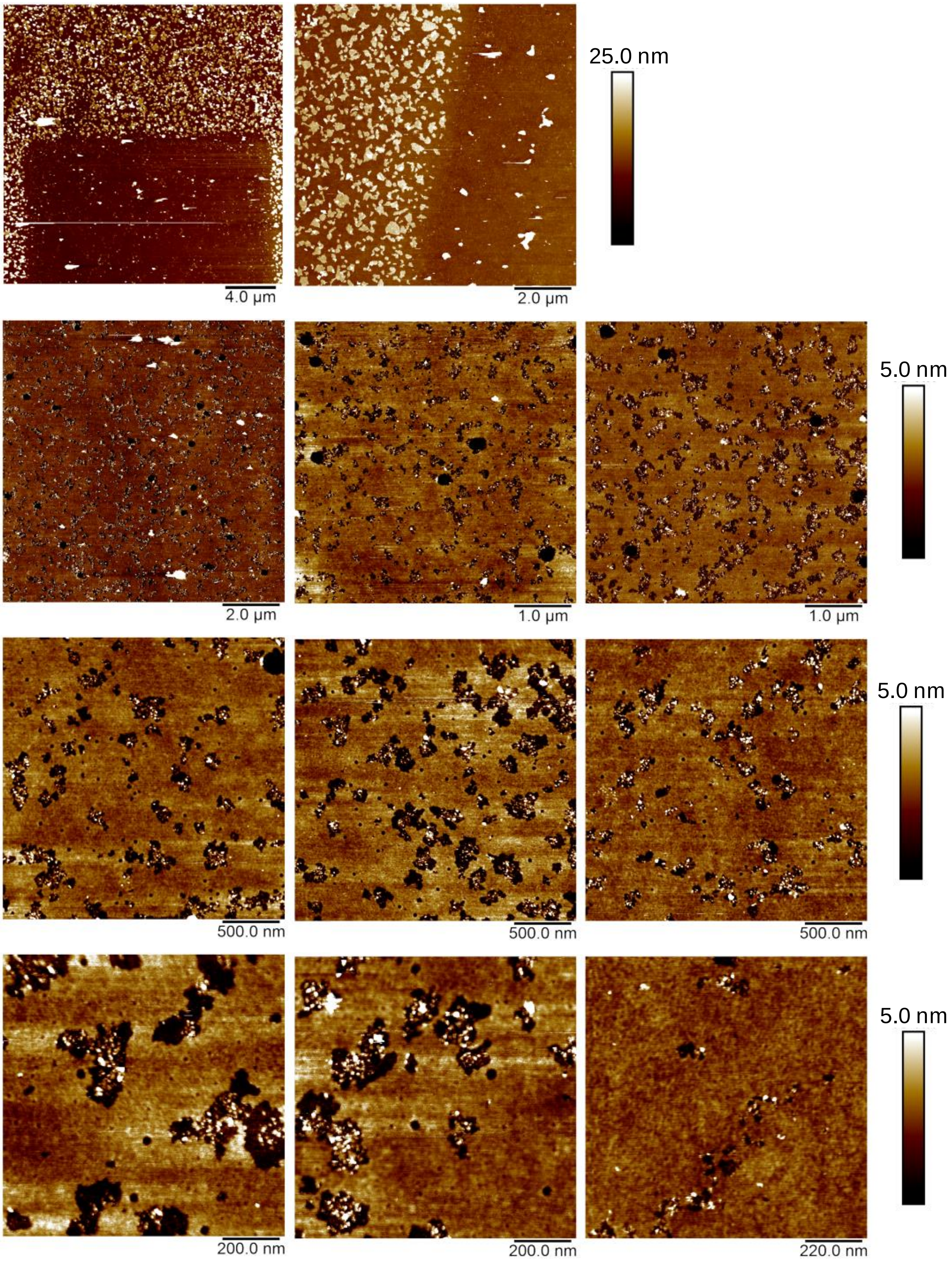

Figure S11: Additional AFM height topographs of hybrid membranes (standard protocol of 1:3 thylakoids-to-DOPC backfilled into a polymerized lipid template). Scale bars are shown for each row of images.

15. Additional spectroscopy data: lack of specificity in the photochemical assays

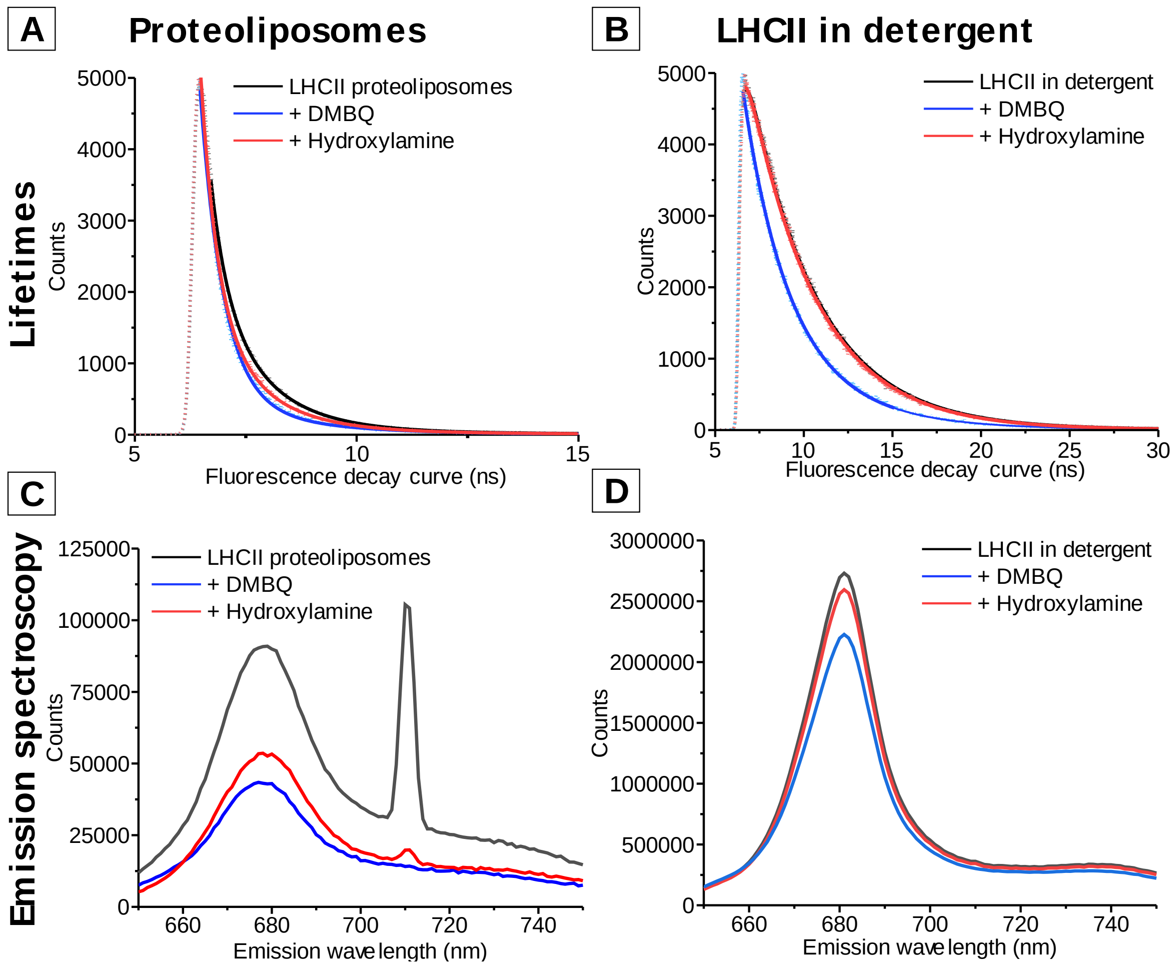

Figure S12: Cuvette-based steady-state and time-resolved fluorescence spectroscopy data of LHCII proteoliposomes (containing 10% w/w LHCII) and LHCII suspended in detergent micelles (in 0.3% α-DDM) in a buffer of 40 mM NaCl 20 mM HEPES pH 7.5. All measurements were performed in 10 × 10 mm quartz cuvettes which were stirred with a magnetic stir bar to mix the solutions. **(A)** Fluorescence lifetime measurements of LHCII proteoliposomes, showing the decrease in fluorescence lifetime upon the addition of DMBQ, and the subsequent recovery of the fluorescence lifetime after the addition of hydroxylamine. **(B)** Fluorescence lifetime measurements of LHCII in detergent, showing the same effects of DMBQ and hydroxylamine as observed in proteoliposomes. **(C) & (D)** Emission spectroscopy plots for LHCII proteoliposomes and LHCII in detergent respectively. In both plots, there is a decrease in fluorescence emission after the addition of DMBQ, followed by the partial or full recovery after the addition of hydroxylamine. This evidence, combined with FLIM measurements (see figure 8) suggest that these photochemical assays, are not specific to the electron transfer photochemistry of PSII.

This cuvette-based fluorescence spectroscopy was performed using an Edinburgh Instruments FLS980 system equipped with dual excitation monochromators and dual

emission monochromators. Samples were maintained at 20 °C and gently stirred using a thermoelectrically-cooled cuvette-holder with magnetic stirring. For steady-state emission spectra, excitation was at 473 nm and emission collected from 500-800 nm (2 nm and 1 nm bandwidth excitation and emission slits, respectively). Time-resolved fluorescence lifetime measurements used a 473 nm pulsed diode laser for excitation (0.5 MHz repetition rate and minimized power) and emission was collected at 681 nm with 10 nm bandwidth emission slits.

16. Control measurements to optimize FLIM acquisitions: selection of an excitation fluence which minimizes singlet-singlet annihilation artefacts

We wanted to minimize any risk of artefacts in fluorescence lifetime in FLIM measurements of photosynthetic proteins, which are known to occur if excitation power is set at too high a level.[2] [9] To confirm this, we varied the excitation power over a range expected to be above and below the level where singlet-singlet annihilation effects may occur. Figure S12 shows fitted lifetimes from FLIM data acquired for a range of photosynthetic samples (both extracted thylakoids and LHCII-only samples). For each sample, multiple different areas were imaged using excitation powers. The excitation power was modulated using a combination of ND filters and an adjustable microblade that obstructs part of the laser beam. The average power readout is provided in arbitrary units within in the SymPhoTime software, which is then converted into SI units using a linear power conversion relationship for each laser wavelength. The pulse FHWM was maintained at a minimal value (<90 ps) by setting the lasing voltage to the minimum threshold required to power the lasers. The pulse repetition rate was set to 10 MHz, such that the fluorophore could fully decay before the next pulse to avoid photon pile-up artefacts. For all lasers, we estimate the laser spot diameter to be ~800nm, allowing us to calculate the peak power per area (fluence) delivered to each sample.

The graph in Figure S12 shows how the average lifetime, <τ>, of each sample changes with the applied fluence (note: <τ> is on a logarithmic scale). In all samples, <τ> remains approximately constant at low fluences (below 0.01 mJ/cm^2), before exponentially decreasing above 0.01 mJ/cm^2 (the unacceptable fluence range is highlighted in red). In order to minimize the collection time required, whilst still maintaining a good signal and an acceptable signal to noise, we selected the upper end of the acceptable fluence range (0.01 mJ/cm^2) to be used for the FLIM acquisition of subsequent samples.

All the lasers share the same ND filter and microblade aperture, however, due to the differences in beam shape and beam diameter, the microblade cut off affects all three lasers differently. Therefore, when operating in Pulsed Interleaved Excitation mode (PIE) mode, setting the laser fluence for the photosynthetic excitation indirectly affected the laser power applied to the Texas Red change. The calculated laser fluences that we used in the current study are shown for 485/640 PIE (combined with the 485/640 dichroic mirror) and for 560/640 PIE (using the 561/640 dichroic mirror) in Table S6 and S7, respectively.

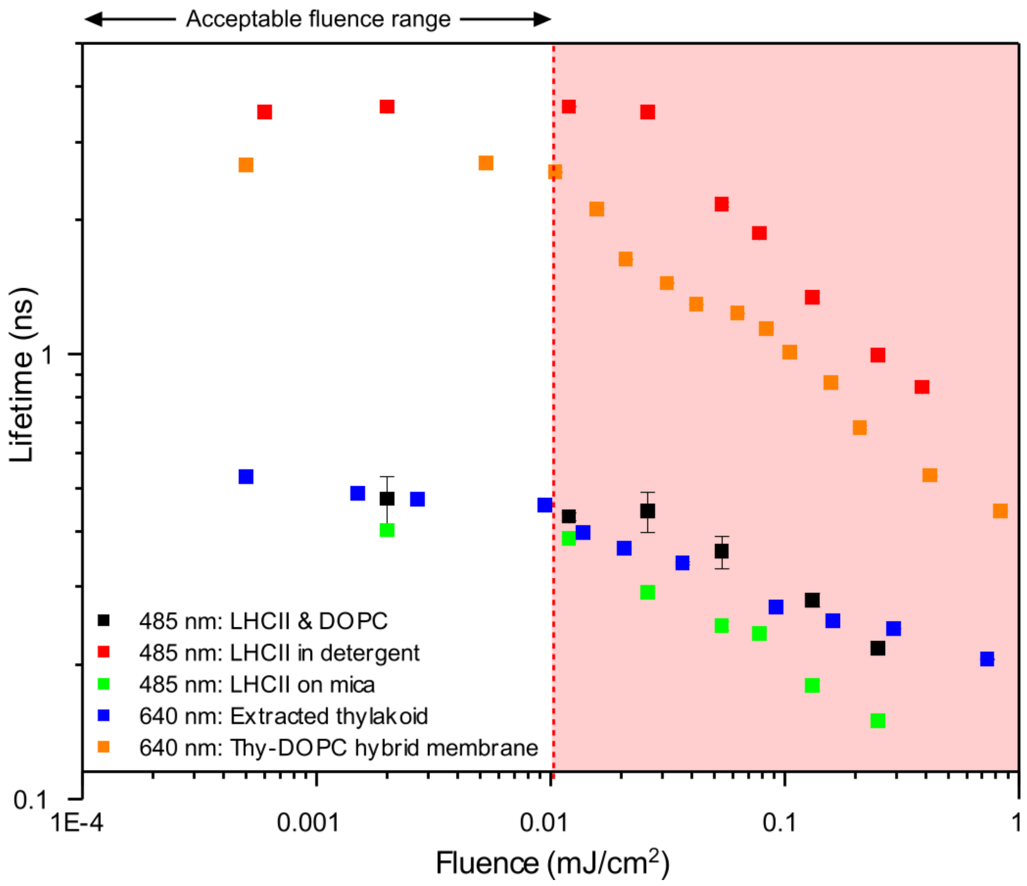

Figure S13: Average fluorescence lifetime versus excitation fluence across multiple photosynthetic samples, highlighting the acceptable fluence range where lifetime artefacts are minimal.

Wave-length	Energy per photon	Laser spot diam.	Rep. rate	Pulse FWHM	Average laser power output	Average intensity	Peak power	Fluence	Fluence after objective
(nm)	(J)	(nm)	(MHz)	(ps)	(W)	(W/cm^2)	(J/s)	(mJ/cm^2)	(mJ/cm^2)
485	4.10×10^{-19}	800	10	90	1.50×10^{-6}	2.98×10^{2}	1.67×10^{-3}	0.030	0.025
640	3.10×10^{-19}	800	10	70	6.85×10^{-7}	1.36×10^{2}	7.61×10^{-4}	0.014	0.012

Table S8: Calculations for the average laser fluence for the FLIM operating in 485/640 PIE mode using the 485/640 dichroic mirror. The 640 nm laser pulse is specifically targeted for the chlorophyll fluorescence, and as such we chose to maintain a low fluence where singlet-single annihilation effects are minimal. This configuration was used when acquiring both the "Diyne-PC FLIM channel" and "Chlorophyll FLIM channel" (main text Figure 4 and suporting information Figure S8).

Wave-length	Energy per photon	Laser spot diam.	Rep. rate	Pulse FWHM	Average laser power output	Average intensity	Peak power	Fluence	Fluence after objective
(nm)	(J)	(nm)	(MHz)	(ps)	(W)	(W/cm^2)	(J/s)	(mJ/cm^2)	(mJ/cm^2)
560	3.55×10^{-19}	800	10	70	1.23×10^{-6}	2.45×10^{2}	1.76×10^{-3}	0.024	0.021
640	3.10×10^{-19}	800	10	90	6.85×10^{-7}	1.36×10^{2}	7.61×10^{-4}	0.014	0.012

Table S9: Calculations for the average laser fluence for the FLIM operating in 560/640 PIE mode using the 560/640 dichroic mirror. The 640 nm laser pulse is specifically targeted for the chlorophyll fluorescence, and as such we chose to maintain a low fluence where singlet-single annihilation effects are minimal. This configuration was used when acquiring both the "Texas Red FLIM channel" and "Chlorophyll FLIM channel" (supporting information Figure S5).

16. Confirmation of the validity of lifetime measurements made in the absence of an O2 scavenging enzyme

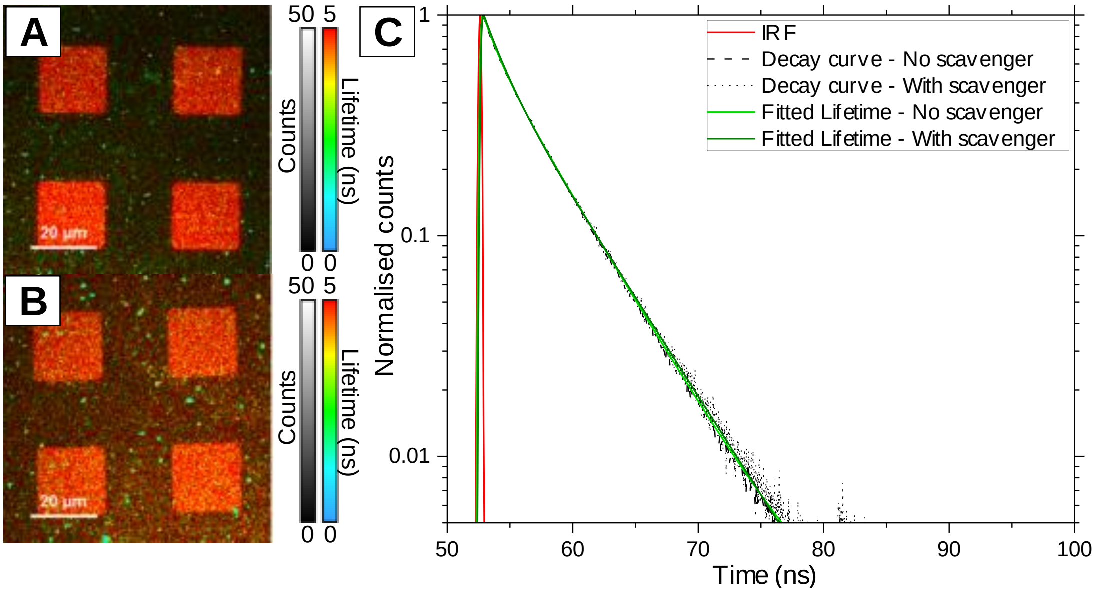

Figure S14: (A) Hybrid membrane that has been prepared and imaged as normal, as described in the main text. **(B)** A hybrid membrane prepared as usual, but subsequently washed into a buffer that has been sparged with dry N_2 gas and contains an O_2 scavenging enzyme system (2.5 mM protocatechuic acid and 50 nM protocatechuate-3,4-dioxygenase

from *Pseudomonas* species).[10] After washing, the sample was allowed to stand for >1 hr at room temperature before imaging, to allow for full removal of any dissolved O_2 from the sample solution. (**C)** Comparison of fluorescence decay curves between the samples with or without the O_2 scavenger. After addition of the O_2 scavenger, there is no significant change in fluorescence lifetimes and only a minimal change in the fluorescence intensity (increased background on the template area), therefore, we can be confident that any dissolved O_2 in the prepared buffers has little effect on our hybrid membrane system.

Supplementary References

[1] a) A. M. Hancock, S. A. Meredith, S. D. Connell, L. J. C. Jeuken, P. G. Adams, *Nanoscale* **2019,** *11*, 16284-16292; b) P. G. Adams, C. Vasilev, C. N. Hunter, M. P. Johnson, *Biochim. Biophys. Acta, Bioenerg.* **2018,** *1859*, 1075-1085; c) B. van Oort, A. van Hoek, A. V. Ruban, H. van Amerongen, *FEBS Lett.* **2007,** *581*, 3528-3532; d) M. Wentworth, A. V. Ruban, P. Horton, *Biochem.* **2004,** *43*, 501-509.

[2] S. Caffarri, T. Tibiletti, R. C. Jennings, S. Santabarbara, *Curr Protein Pept Sc.* **2014,** *15*, 296-331.

[3] a) J. Chmeliov, A. Gelzinis, E. Songaila, R. Augulis, C. D. P. Duffy, A. V. Ruban, L. Valkunas, *Nature Plants* **2016,** *2*, 7; b) M. P. Johnson, A. V. Ruban, *J. Biol. Chem.* **2009,** *284*, 23592-23601.

[4] R. D. Hills, N. McGlinchey, *J. Comput. Chem.* **2016,** *37*, 1112-1118.

[5] R. Standfuss, A. C. T. van Scheltinga, M. Lamborghini, W. Kuhlbrandt, *EMBO J.* **2005,** *24*, 919-928.

[6] I. Langmuir, *J. Am. Chem. Soc.* **1918,** *40*, 1361-1403.

[7] P. Bao, M. L. Cartron, K. H. Sheikh, B. R. G. Johnson, C. N. Hunter, S. D. Evans, *Chem. Commun.* **2017,** *53*, 4250-4253.

[8] M. P. Johnson, C. Vasilev, J. D. Olsen, C. N. Hunter, *Plant Cell* **2014,** *26*, 3051-3061.

[9] V. Barzda, V. Gulbinas, R. Kananavicius, V. Cervinskas, H. van Amerongen, R. van Grondelle, L. Valkunas, *Biophys. J.* **2001,** *80*, 2409-2421.

[10] C. E. Aitken, R. A. Marshall, J. D. Puglisi, *Biophys. J.* **2008,** *94*, 1826-1835.
